# Supplementary material for: Probing strigolactone perception mechanisms with rationally designed small-molecule agonists stimulating germination of root parasitic weeds
Source: Nat Commun. 2022 Jul 9;13:3987. doi: 10.1038/s41467-022-31710-9 (PMC9271048; doi:10.1038/s41467-022-31710-9)
Supplement: Supplementary file 1 — Supplementary Information [file 41467_2022_31710_MOESM1_ESM.pdf]

Supplementary Information

Probing strigolactone perception mechanisms with rationally designed small-molecule agonists stimulating germination of root parasitic weeds

Dawei Wang[1,5], Zhili Pang[1,5], Haiyang Yu[2], Benjamin Thiombiano[3], Aimee Walmsley[3], Shuyi Yu[1], Yingying Zhang[1], Tao Wei[1], Lu Liang[1], Jing Wang[4], Xin Wen[1], Harro J. Bouwmeester[3*], Ruifeng Yao[2]*, Zhen Xi[1]*

[1]State Key Laboratory of Elemento-Organic Chemistry and Department of Chemical Biology, National Pesticide Engineering Research Center, Collaborative Innovation Center of Chemical Science and Engineering, College of Chemistry, Nankai University, Tianjin 300071, P. R. China

[2]State Key Laboratory of Chemo/Biosensing and Chemometrics, Hunan Provincial Key Laboratory of Plant Functional Genomics and Developmental Regulation, College of Biology, Hunan University, Changsha 410082, P. R. China

[3]Swammerdam Institute for Life Sciences (SILS), University of Amsterdam, Science Park 904, 1098 XH Amsterdam, Netherlands.

[4]State Key Laboratory of Natural and Biomimetic Drugs, School of Pharmaceutical Sciences, Peking University, Beijing 100871, P. R. China

[5]These authors contributed equally to this work: Dawei Wang and Zhili Pang.

*Corresponding authors. Email: H.J.Bouwmeester@uva.nl (H.J.B.); ryao@hnu.edu.cn (R.Y.); zhenxi@nankai.edu.cn (Z.X.).

Supplementary Methods

Crystallization

The protein solution was concentrated to 7.0 mg/mL for crystallization. The crystallization trial was performed by the sitting-drop vapor diffusion method at 20 °C in 48-well plates with commercially available kits (Hampton Research and Rigaku). The crystals were obtained in a reservoir solution containing 0.1 M Tris-HCl pH 8.5, 0.1 M $MgCl_2$, 25% (*w*/*v*) PEG 4000 in sitting-drop vapor diffusion method. After optimization, high-quality crystals were obtained in a reservoir solution containing 0.1 M Tris-HCl pH 8.0, 0.1 M $MgCl_2$, 21% (*w*/*v*) PEG 4000 in the hanging-drop method. Crystals were picked up and transferred into a reservoir solution containing 35% (*v*/*v*) glycerol before they were flash-frozen in liquid nitrogen.

X-ray diffraction data were collected on beamline BL-19U1 (λ = 0.9789) at Shanghai Synchrotron Radiation Facility (SSRF) using a CCD Pilatus CBF detector which was carried out at 100 K. A total of 360 images were collected at a crystal-to-detector distance of 300 mm with 0.3 s exposure for every 0.5° oscillation frame. The Data were indexed, integrated, and scaled using HKL-2000[1]. Molecular replacement was performed by using the structure of ShHTL5 (PDB entry 5Z7Y). Coot was used to manually fit the protein model and the CCP4 program was performed for model refinement[2]. Finally, the program PROCHECK was used to check the model. We got the final result R_{factor} of 18.34% and a R_{free} of 23.52%. The structure has been deposited to the RCSB Protein Data Bank (PDB) with the accession code 6A9D. The data collection and processing statistics are summarized in Supplementary Table 2.

Molecular simulation studies

Molecular docking. The 3D structures of (*R*)- and (*S*)-**4a** were constructed by Sybyl 6.9 (Tripos Inc.,

St. Louis, MO), and the molecules were subjected to energy minimization at a gradient of 1.0 kcal/mol with a delta energy change of 0.05 cal/mol. The crystal structure (PDB id: 6A9D) of ShHTL7 was used as the docking receptor and the binding site was defined based on the catalytic triad. The ligands and receptors were prepared by Mgltools 1.5.6[3]. Autodock 4.2 was used for docking ligands into the active site of ShHTL7[3]. The conformational search of ligands in the active site was performed by Lamarckian genetic algorithm (LGA). A total of 500 runs were launched for each ligand. Other parameters used for the docking studies were set to the default values as recommended by Autodock. After docking, the results were clustered, and the docking results were selected based on the docking score as well as referred to the simulated binding mode of GR24 in rice D14 (PDB entry 5DJ5)[4].

MD simulation and P_{PC} analysis. MD simulations were performed by using Amber 14 with the ff14SB force filed[5]. The force-field parameters of ligands were generated by the Gaussian 03 program and the Antechamber program[6]. Each complex was solvated with TIP3P water molecules in an 8.0 Å truncated octahedral box and Na^+ ions were added to neutralize each complex system.

Before MD simulations, a series of energy minimizations of each complex system was performed with particle mesh Ewald, periodic boundary conditions, and the SHAKE method. After that, the system was gradually heated from 0 to 300K in 50 ps, and a 50 ps equilibrating calculation was executed at 1 atm and 300K. The 1000 ns MD simulations of each system were performed. The snapshots of the systems were recorded every 1 ps. The $D_{5'C\text{-}OG}$, $D_{NE2\text{-}HG}$, $D_{OD1\text{-}HD1}$, $D_{OD2\text{-}HD1}$, $D_{2'C\text{-}NE2}$, and $D_{NE2\text{-}HG}$ were analyzed using the cpptraj module.

The distances of $D_{5'C\text{-}OG}$ and $D_{NE2\text{-}HG}$ in the MD simulations of each system were converted to the sets of discrete data points of $D_{5'C\text{-}OG}$-$D_{NE2\text{-}HG}$ pairs to the conformation probability density function (CPDF), which denoted as $Z(A,D)$[7]. The CPDF of each system was constructed using the following steps: first, the plane of $D_{5'C\text{-}OG}$-$D_{NE2\text{-}HG}$ was divided into square bins with 0.1 Å length and

0.1 Å width; second, the number of data points of each bin was counted; third, the density of each bin is calculated using the number of $D_{5'C\text{-}OG}$-$D_{NE2\text{-}HG}$ data points fall in the corresponding bin being divided by the total number of the distance-distance data points; last, the $Z(A,D)$ (shown in Equation 1) was constructed by fitting the data with the two-dimensional Gaussian function using the non-linear surface fitting methods in Origin Lab.

$$Z(A,D) = C_{\exp}\left\{-\frac{1}{2}\left(\frac{A-\mu_A}{\sigma_A}\right)^2 - \frac{1}{2}\left(\frac{D-\mu_D}{\sigma_D}\right)^2\right\} \quad \text{(Equation 1)}$$

In this equation, Z is the conformation probability density of $D_{5'C\text{-}OG}$ and $D_{NE2\text{-}HG}$ in the calculation system. μ is the mean of the normal distribution. σ is the standard deviation.

The P_{PC} of each system was calculated using the bivariate integration of the corresponding CPDF over the interval 1.5–3.8 Å for $D_{5'C\text{-}OG}$, and 1.6–3.5 Å for $D_{NE2\text{-}HG}$ with Equation 2 using Matlab software (The Math Works, Natick, MA).

$$P_{PC} = \int_{A_2}^{A_1}\int_{D_2}^{D_1} C_{\exp}\left\{-\frac{1}{2}\left(\frac{A-\mu_A}{\sigma_A}\right)^2 - \frac{1}{2}\left(\frac{D-\mu_D}{\sigma_D}\right)^2\right\} dA dD \quad \text{(Equation 2)}$$

The final P_{PC} value of each system was the averaged data of every 200 ns in the MD simulation. The structure figures were analyzed by PYMOL software[8].

DFT calculations

The structures of (*S*)-**4a** were constructed and optimized by Sybyl 6.9 (Tripos Inc., St. Louis, MO). DFT calculations of (*S*)-**4a** were performed by Gaussian 09 program package at b3lyp/6-31g(d) level[9]. The results were analyzed by GaussView 5.0.

Determination of the Inhibition Model

For Lineweaver-Burk plot analysis, the competitive inhibition of ShHTL7 by compounds **1**, **2g**, and **2h** was performed by using YLG as the substrate at 25 °C. The excitation and emission

wavelengths of YLG are 480 nm and 520 nm, respectively. The testing compounds and YLG were dissolved in DMSO and prepared as stock solutions just before use. A total of l00 μL reaction mixture, which consisted of 3.6 μg recombinant ShHTL7 protein, 1–5 μM YLG in reaction buffer (100 mM HEPES, 150 mM NaCl, pH 7.0), and 0–200 μM compounds were added to a 96-well black plate (Thermo). The fluorescence changes of each well at 520 nM were collected using a TECAN GENios Plus microplate reader every 40 second for 30 min. The linear fluorescence increase in 8 min was used for further inspection. The velocity (ν) at various probe concentrations was calculated for subsequent analysis[10]. The substrate and inhibitor concentrations are shown in each figure (Supplementary Fig.7).

Synthetic methods

All chemical reagents were purchased from commercial suppliers (Innochem, Science & Technology Co., Ltd., Beijing, China; J&K Scientific Ltd., Beijing, China) and treated with standard methods before use. CH_2Cl_2 and Et_3N were distilled from CaH just before use; other solvents such as petroleum ether (bp 60–90 °C), ethyl acetate, *n*-pentane, and ether were purchased from Sinopharm Chemical Reagent Co., Ltd. and used as received. Silica gel (200–300 mesh) for column chromatography (CC) was purchased from Qingdao Haiyang Chemical Co., Ltd., Qingdao, China. Analytical thin-layer chromatography (TLC) was performed on E. Merck Silica gel60 F_{254} precoated Aluminum plates (0.25 mm). Compounds were visualized by spraying 0.05% potassium permanganate aqueous solution on the TLC plates. ^{1}H NMR and ^{13}C NMR spectra were recorded in $CDCl_3$, CD_2Cl_2 or DMSO-d_6 on a Varian Mercury 400 spectrometer, and resonances (δ) are given in ppm relative to tetramethylsilane (TMS) or CD_2Cl_2. High-resolution mass spectra (HRMS) were recorded on a 6520 Q-TOF LC/MS (Agilent, Santa Clara, CA). Optical rotations were determined using a Perkin Elmer 341 polarimeter with $CHCl_3$ as the solvent. The chiral isomers of **3f**, **3g** and **4a** were analyzed by Shimadzu LC-20AD CP-HPLC-08 instruments. Semi-preparative HPLC was performed by using YMC-1 (K-PREP-K-100) instruments.

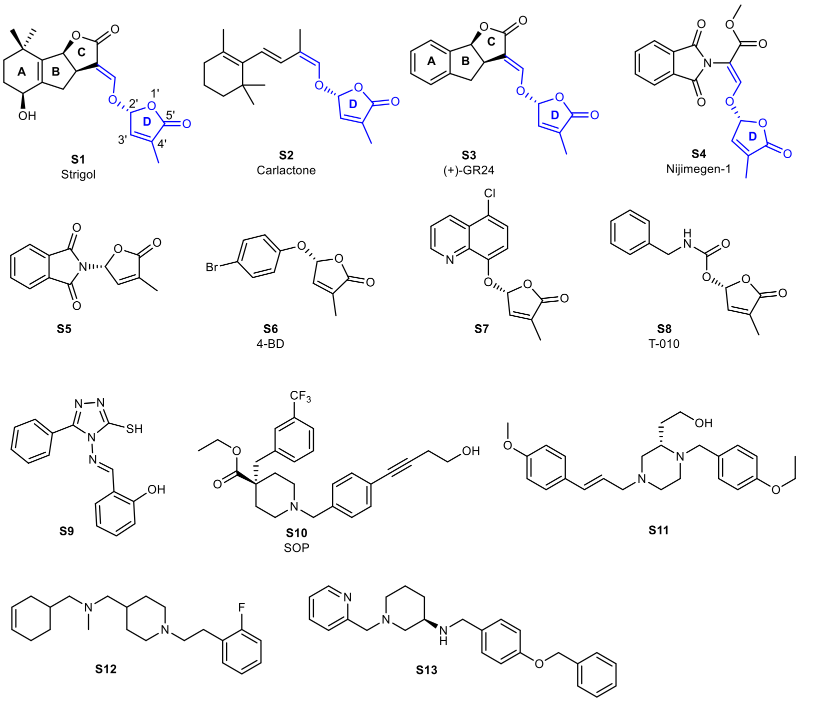

Supplementary Fig. 1. **Chemical structures of 13 representative search query molecules.**

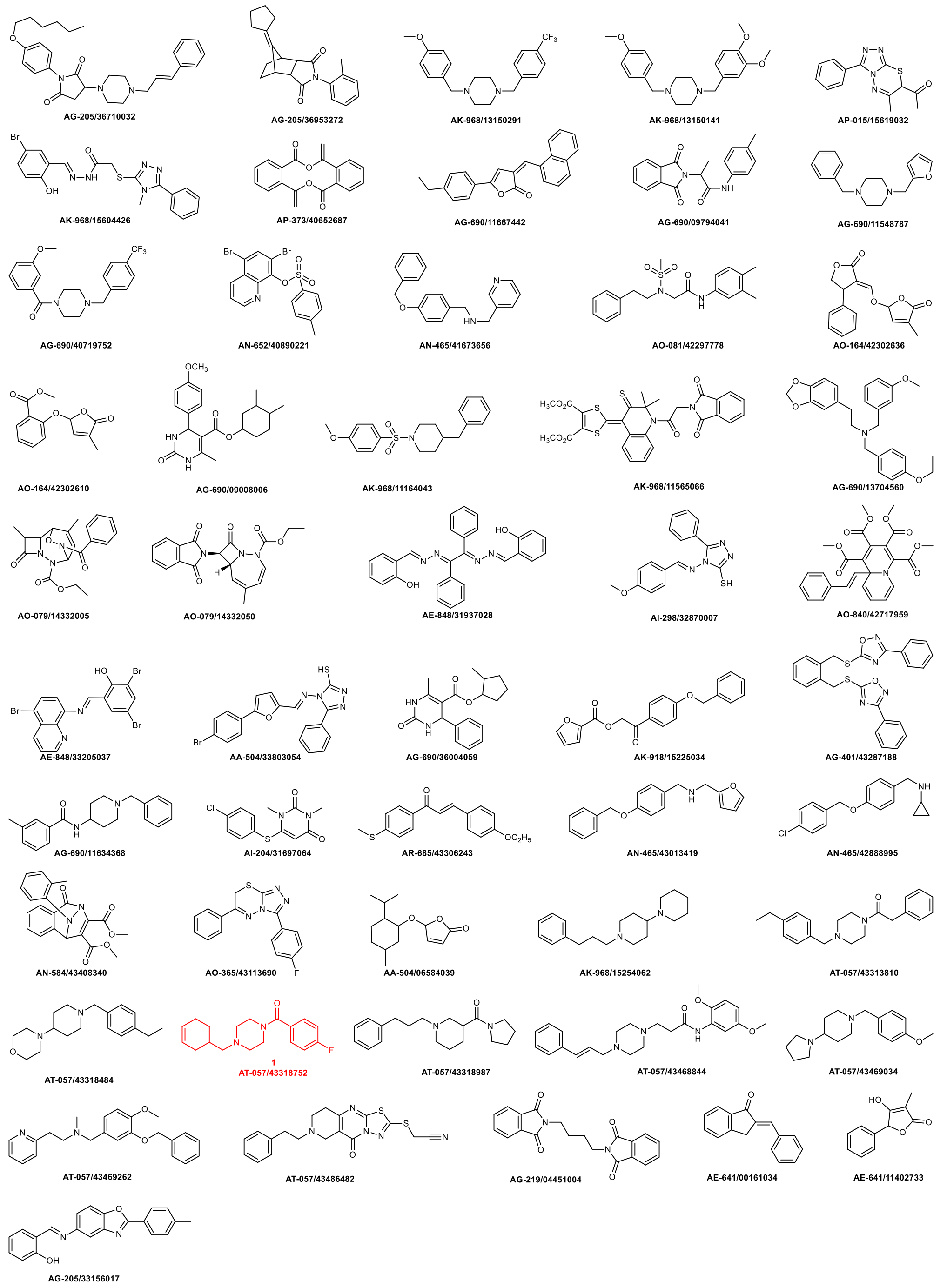

Supplementary Fig. 2. Structure of the 51 candidates from the Specs database in the first round of virtual screening. The structure of hit compound **1** (AT-057/43318752) is highlighted in red color.

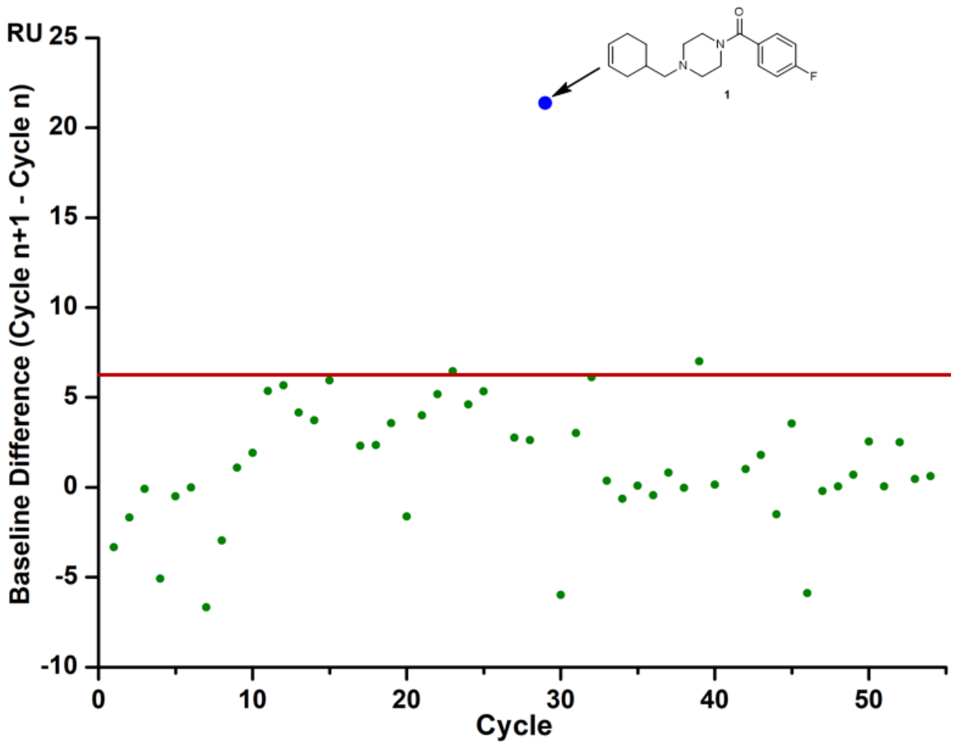

Supplementary Fig. 3. **The binding levels of 51 compounds screened by the surface plasmon resonance (SPR) method against ShHTL7 at 50 μM**. Positive compounds with RU equal or above 6 were obtained using the kinetics/binding affinity analysis in the Biacore evaluation software (T200 Version 2.0).

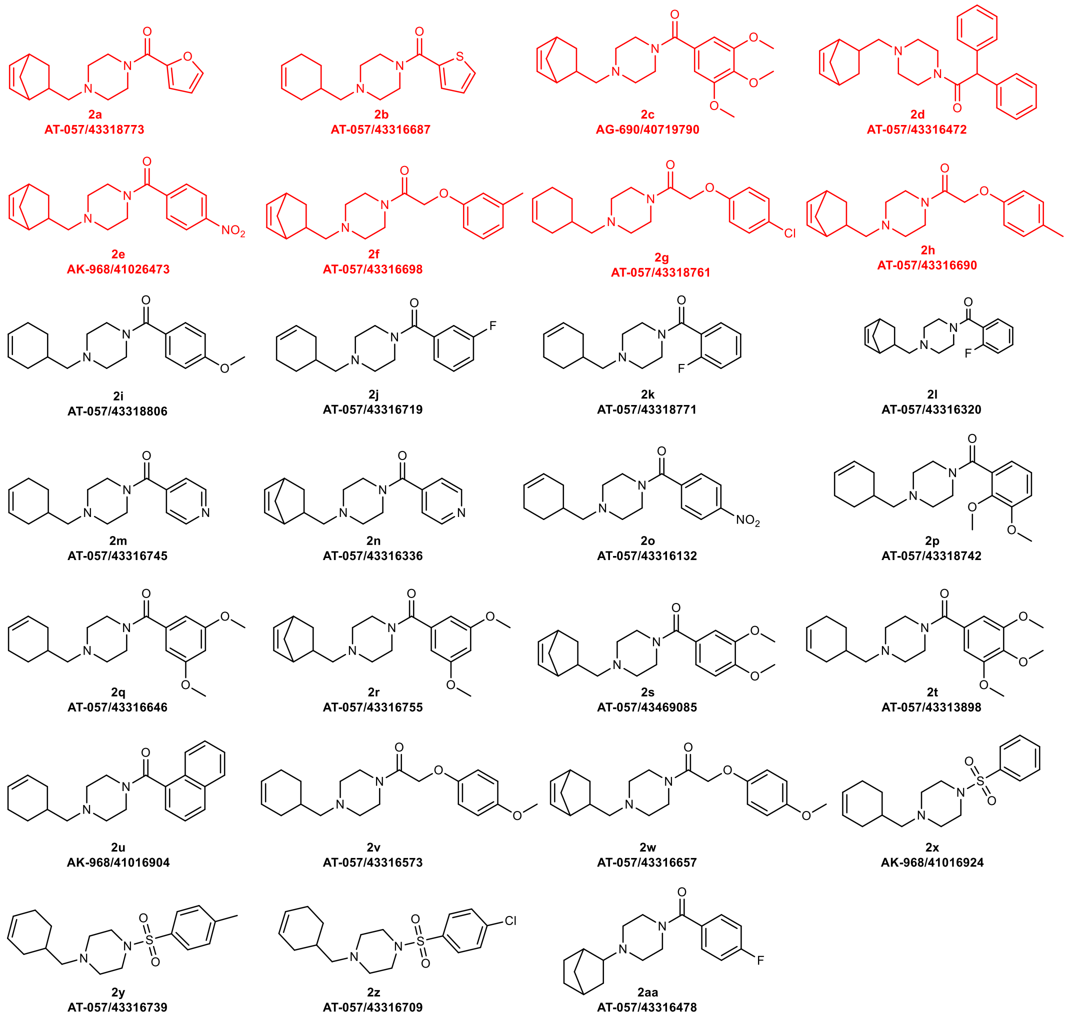

Supplementary Fig. 4. Structure of the 27 candidates from the Specs database in the second round of virtual screening. The structures of compounds with higher K_D values than the hit compound **1** in the first round of screening are highlighted in red color.

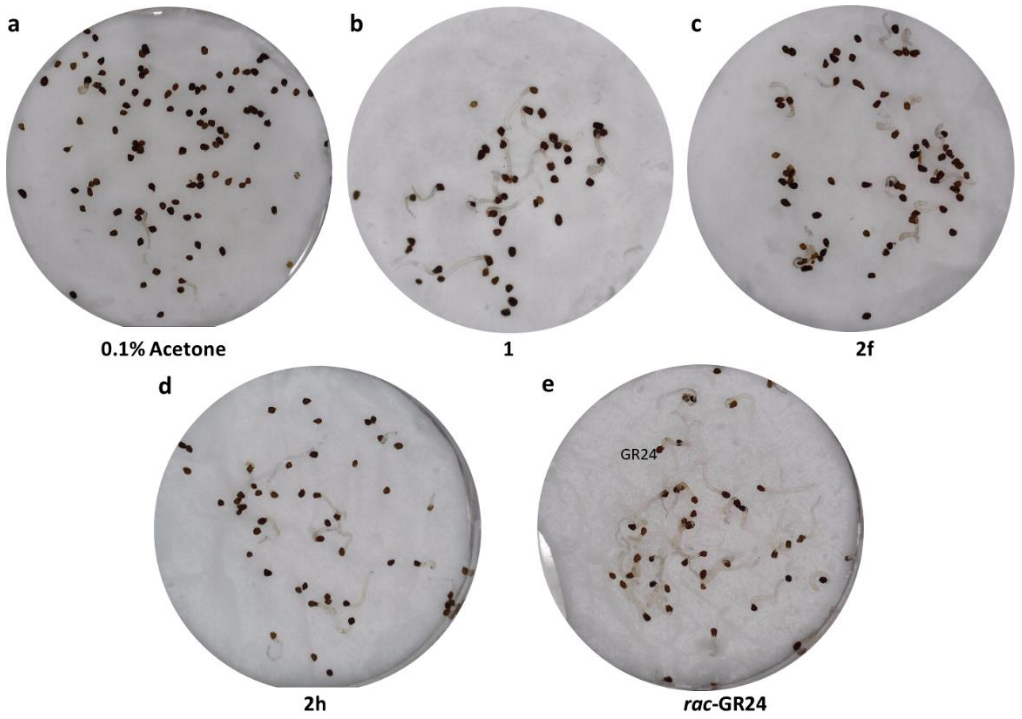

Supplementary Fig. 5. **Germination of *P. aegyptiaca* seeds after 7 days of treatment with tested compounds at 10 μM**. **a**, control, 0.1% acetone solution. **b**, compound **1**. **c**, compound **2f**. **d**, compound **2h**. **e**, *rac*-GR24. The figures are representative of three independent experiments.

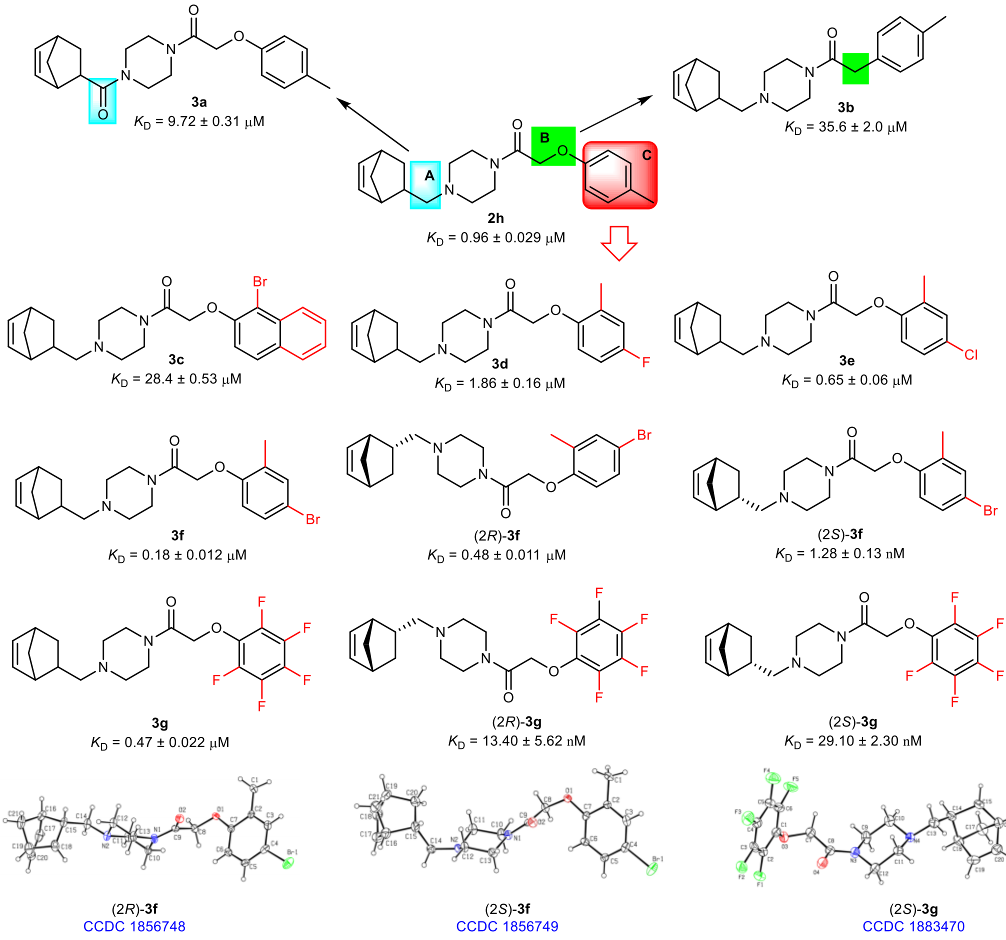

Supplementary Fig. 6. Structure-activity relationship studies on hit compound 2h. The K_D values of the binding affinities with ShHTL7 are shown below each molecule. Three major modifications marked with different colors were made on lead compound **2h**. Modifications of **2h** on site A are shown in cyan, modifications of **2h** on site B are shown in green, and modifications of **2h** on site C are shown in red. The crystal structure of (2*R*)-**3f**, (2*S*)-**3f**, and (2*S*)-**3g** are shown in the ball-and-stick model.

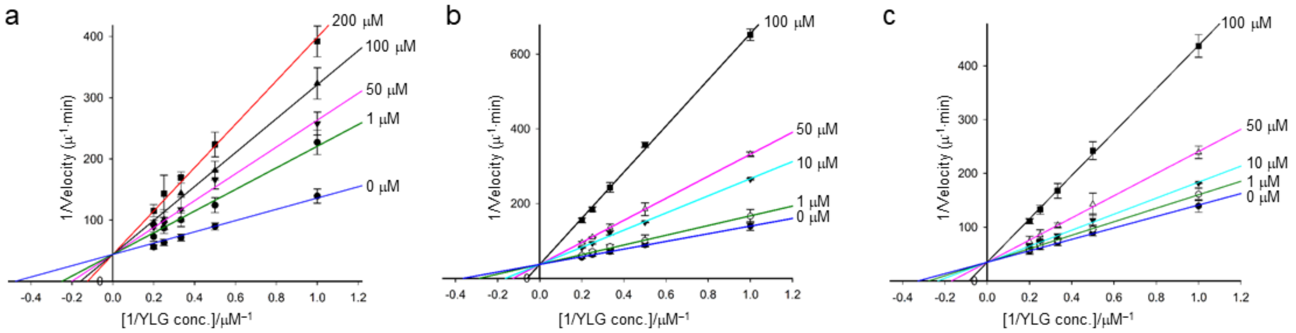

Supplementary Fig. 7. Lineweaver−Burk plots for the inhibition of ShHTL7 by compounds **1** (**a**), **2g** (**b**), and **2h** (**c**). Error bar indicates SD (n = 3 biologically independent experiments).

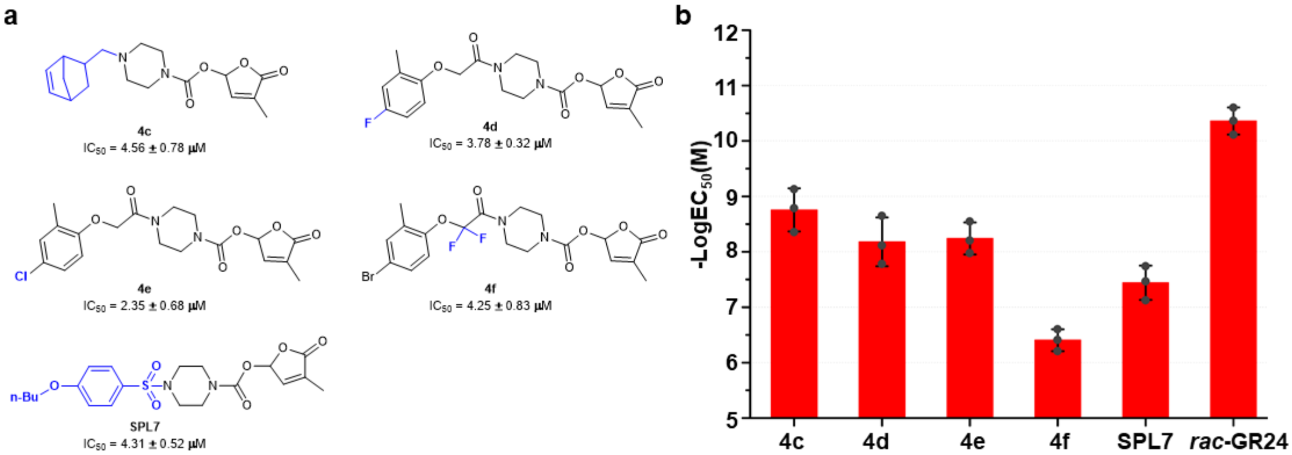

Supplementary Fig. 8. **ShHTL7 competitive inhibition activity in YLG assays and *P. aegyptiaca* germination activity of compounds 4c-4f, SPL7, and *rac*-GR24**. **a**, Structures of **4c-4f**, and **SPL7** and their ShHTL7 competitive inhibition activity, the structure changes relative to compound **4a** are shown in blue color. Data are the means ± SD (n = 3 biologically independent experiments). **b**, The EC_{50} values of **4c-4f**, and **SPL7** in stimulating *P. aegyptiaca* seed germination. The error bar indicates SD (n = 3 biologically independent experiments).

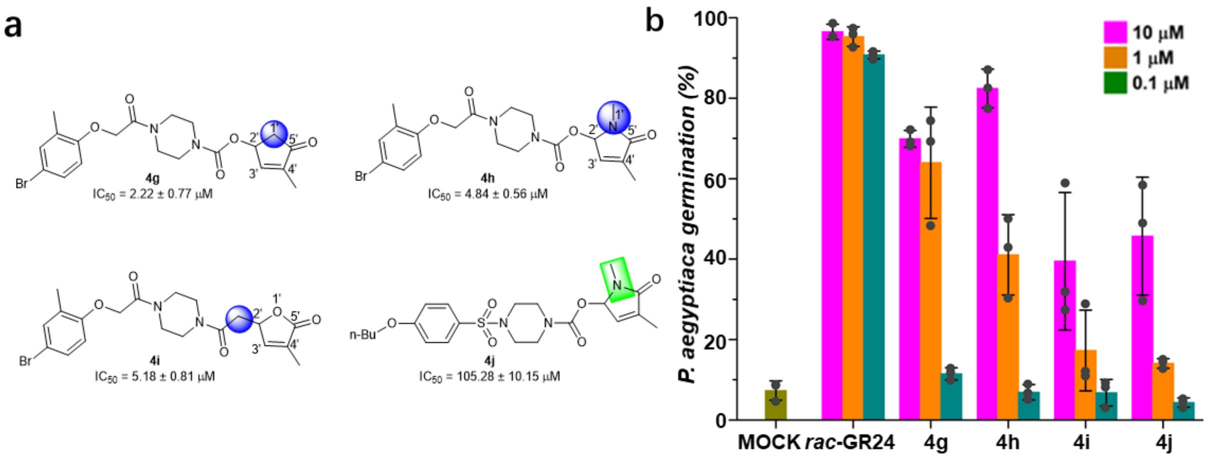

Supplementary Fig. 9. **ShHTL7 competitive inhibition activity with YLG and *P. aegyptiaca* seed germination stimulating activity of compounds 4g-j. a**, Structures of **4g-j** and their ShHTL7 inhibitory activity were shown. The structure changes of **4g-j** relative to compound **4a** are shown in blue color, change of **4k** relative to **SPL7** is shown in green color. Data are the means ± SD (n = 3 biologically independent experiments). **b**, The *P. aegyptiaca* seed germination activity of **4g-j**, and *rac*-GR24. The error bar indicates SD (n = 3 biologically independent experiments).

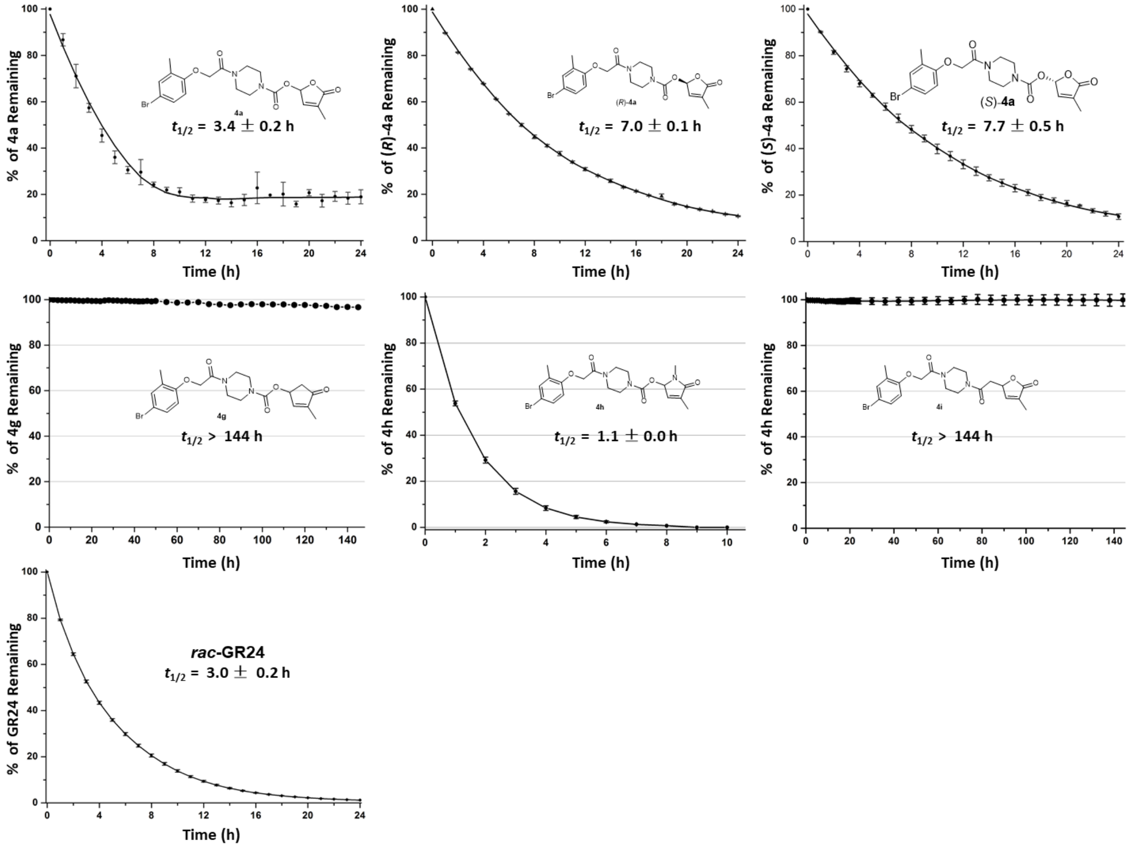

Supplementary Fig. 10. **Chemical reactivity of 4a, (*R*)-4a, (*S*)-4a, 4g-i, and *rac*-GR24 in PBS (10 mmol)/methanol = 3:1 at pH 7.4**. The reactivity tests were performed at 26 ± 1 °C *in vitro*. The error bar indicates SD (n = 3 biologically independent experiments).

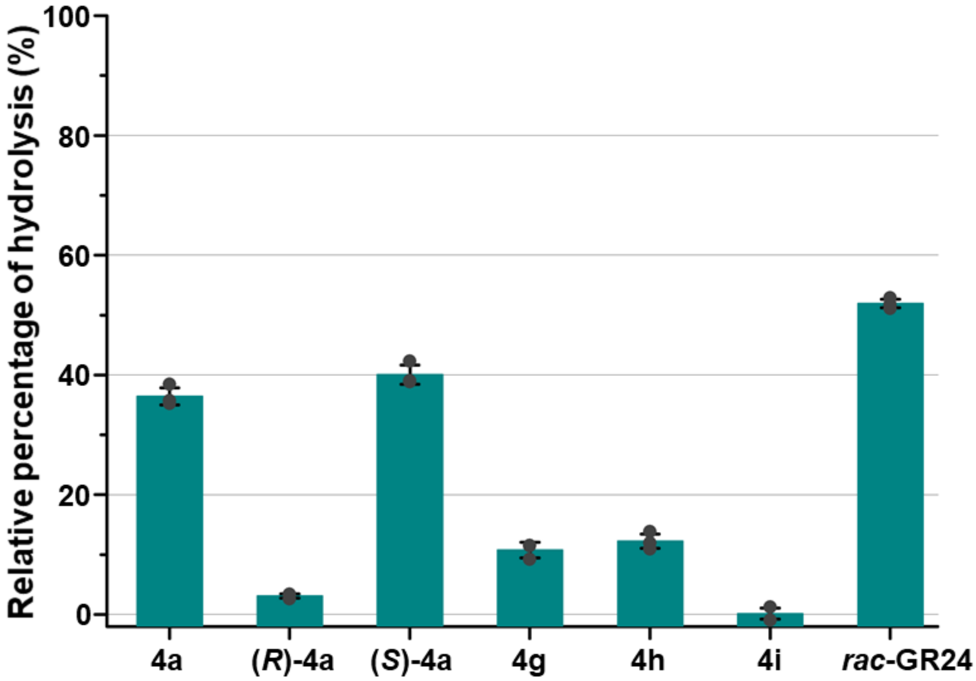

Supplementary Fig. 11. Hydrolysis of 4a, (*R*)-4a, (*S*)-4a, 4g-i, and *rac*-GR24 by ShHTL7 at 30 °C for 30 min. The remaining amounts of compounds in recombinant ShHTL7 protein and buffer solution were determined by HPLC analysis. The relative percentages of hydrolysis were calculated by using the amount of compounds in the buffer as reference. The error bar indicates SD (n = 3 biologically independent experiments).

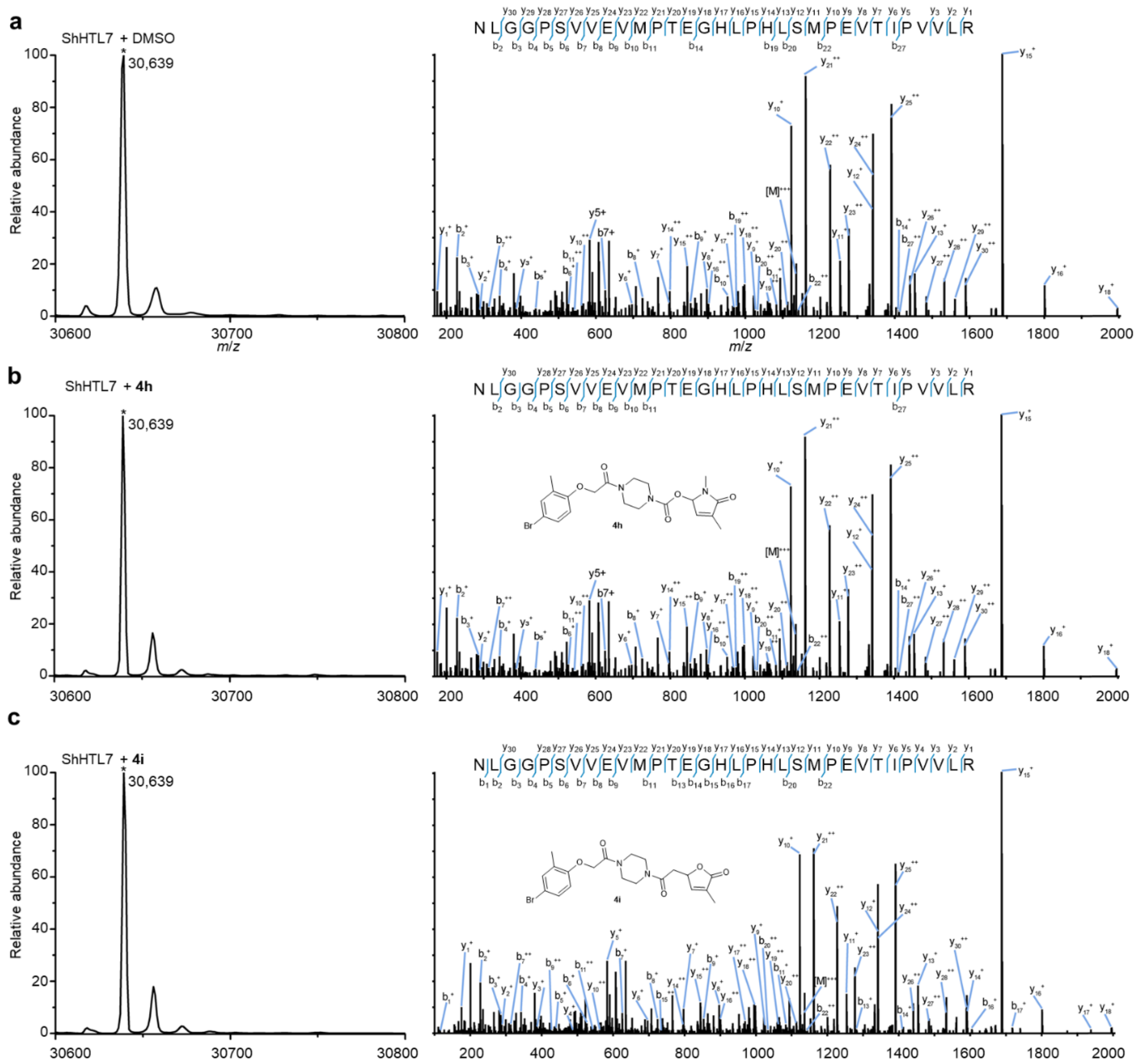

Supplementary Fig. 12. The CLIM modification on ShHTL7 of 4h and 4i was not found from mass spectrum analysis. **a**, MS spectrum of recombinant ShHTL7 protein in assay buffer with 0.1% DMSO were analyzed from nanoACQUITY UPLC-SYNAPT-G2-Si mass spectrometer system (left) and Q-TOF mass spectrometer system (right), respectively. *m*/*z* of recombinant ShHTL7 was determined as 30639. **b**, MS spectrum analysis of recombinant ShHTL7 protein treated with 400 μM **4h**. No modifications were observed, compared to DMSO treated samples in **a**. **c**, MS spectrum analysis of recombinant ShHTL7 protein treated with 400 μM **4i**. No modifications were observed, compared to DMSO treated samples in **a**.

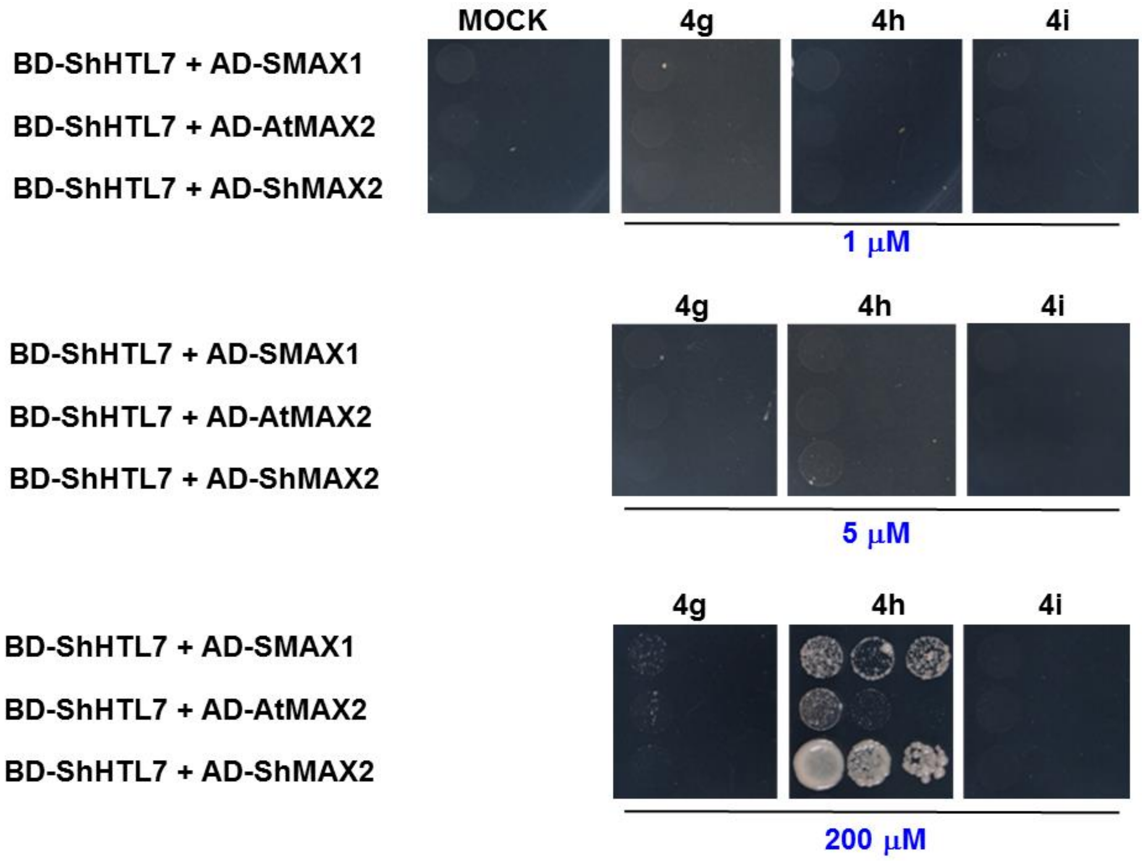

Supplementary Fig. 13. **Effect of 4g-i on the interaction of ShHTL7 with SMAX1, AtMAX2, and ShMAX2.** Yeast two-hybrid assays were performed for ShHTL7 and AtD14 interactions with SMAX1, AtMAX2, ShMAX2, respectively. ShHTL7 and AtD14 were fused to GAL4-BD, SMAX1, AtMAX2, ShMAX2 were fused to GAL4-AD. Serial 10-fold dilutions of yeast cultures were spotted onto a selective growth medium that was supplemented with different concentrations of **4g-i**, respectively. Images show growth after 3 d on SD/-Leu-Trp-His-Ade plates at 30 °C.

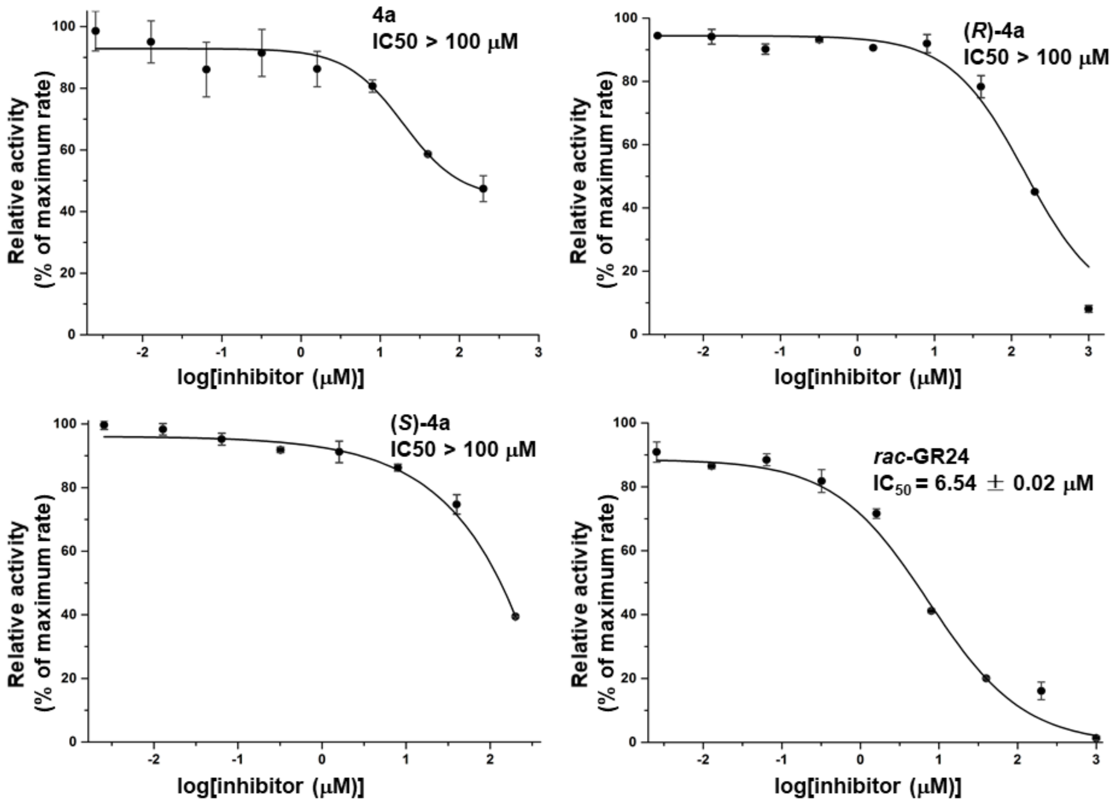

Supplementary Fig. 14. **Competitive inhibition of AtD14 activity curve under the different concentrations of 4a, (*R*)-4a, (*S*)-4a, and *rac*-GR24**. Error bars mean SD (n = 3 biologically independent experiments).

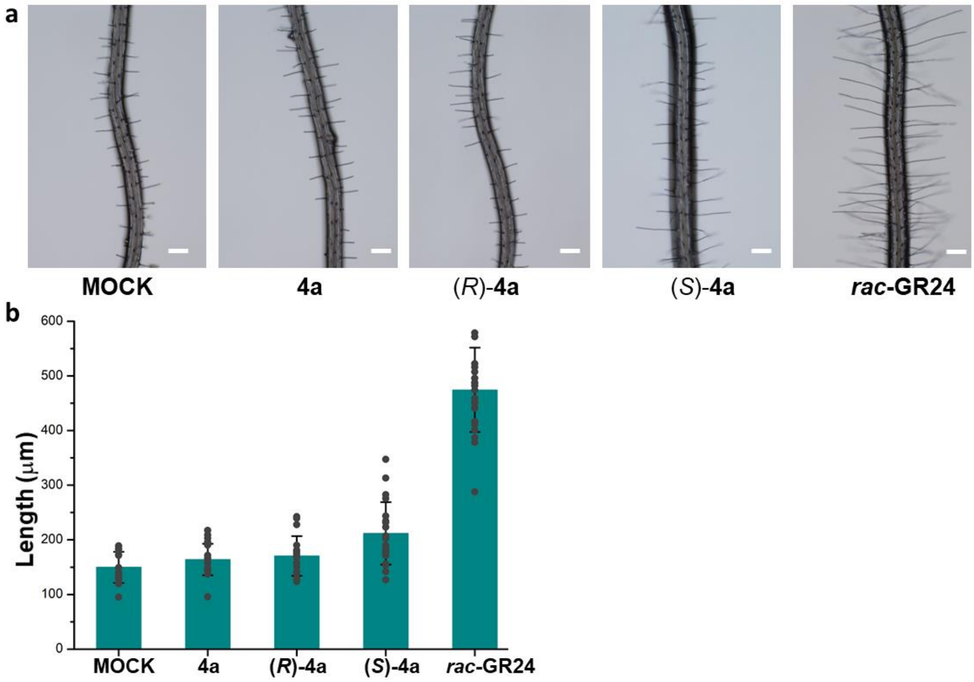

Supplementary Fig. 15. **4a, (*R*)-4a, and (*S*)-4a failed to induce the root hair length in the wild-type *Arabidopsis***. **a**, Representative primary root segments of Col-0 seedlings growing on 1/2 MS medium with or without 1 μM tested chemicals. Scale bar, 200 mm. **b**, The average length of root hair is presented as means ± SD (n = 3 biologically independent experiments).

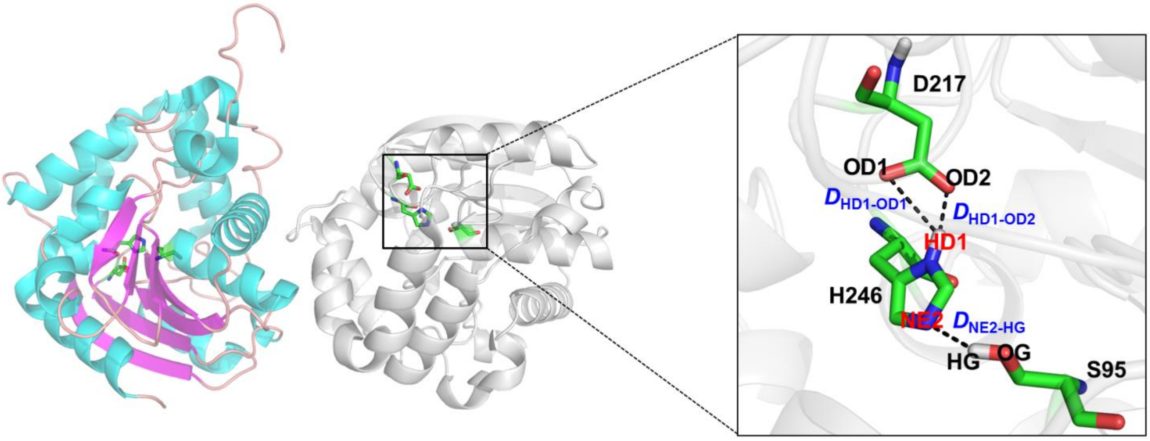

Supplementary Fig. 16. **Overall crystal structure of apo ShHTL7 (PDB entry 6A9D).** The backbones of the dimer are shown in the cartoon. The Ser-Asp-His catalytic triads in the binding pockets are shown in green sticks. The hydrogen atoms of the residues were added by the pdb4amber program of amber14. The hydrogen-bonding interactions of the catalytic triads are shown in black dashed lines. The distance between HD1 of H246 and OD1 of D217 is defined as $D_{\mathrm{HD1\text{-}OD1}}$, and the distance between HD1 of H246 and OD2 of D217 is defined as $D_{\mathrm{HD1\text{-}OD2}}$.

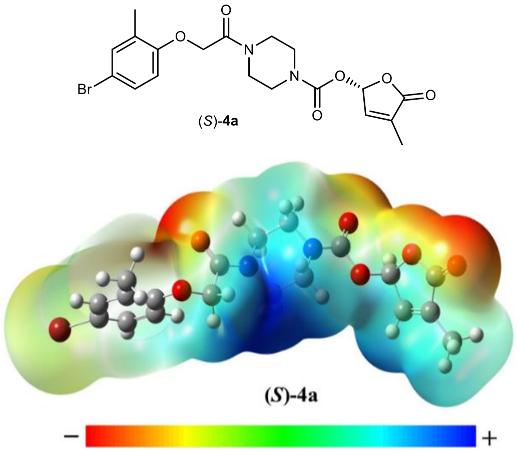

Supplementary Fig. 17. **Electrostatic potential (ESP) surface of compound (*S*)-4a**. The structure of (*S*)-**4a** was constructed using Sybyl 6.9, and the electrostatic potential surface was calculated using Gaussian 09 program package at b3lyp/6-31g(d) level. The red regions represent the negative ESP surface and are easier to donate electrons. The blue regions represent the positive ESP surface and are easier to accept electrons.

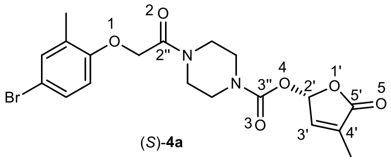

(*S*)-**4a**

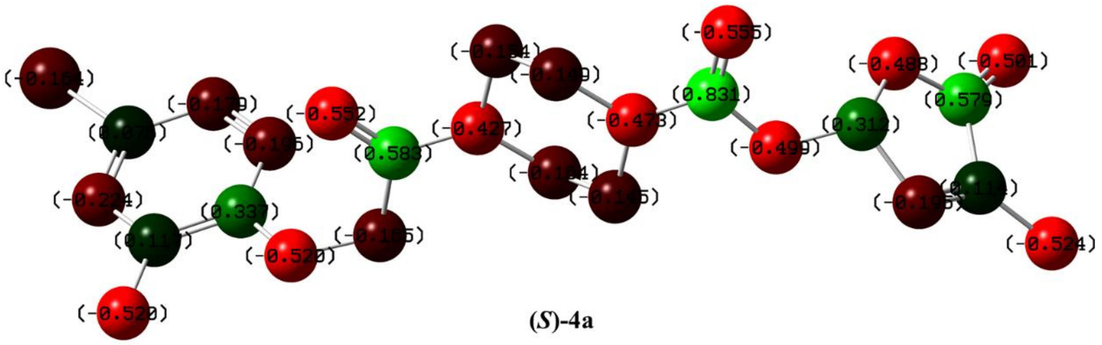

(*S*)-4a

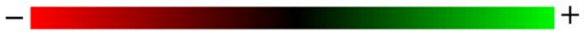

Mulliken atomic charges of (*S*)-4a		
**Atom No.**	**Atom type**	**Mulliken charge**
1	O	−0.520
2	O	−0.552
3	O	−0.555
4	O	−0.499
5	O	−0.501
1′	O	−0.488
**2′**	**C**	**0.312**
3′	C	−0.195
4′	C	0.114
**5′**	**C**	**0.579**

Supplementary Fig. 18. The Mulliken atomic charges (in a.u.) distribution of the heavy atoms of (*S*)-4a. The structures of (*S*)-**4a** were constructed by Sybyl 6.9, and the Mulliken atomic charges were calculated using Gaussian 09 program package at b3lyp/6-31g(d) level. The minus Mulliken charge represents the negative ESP surface and is easier to donate electrons. The plus Mulliken charge represents the positive ESP surface and is easier to accept electrons.

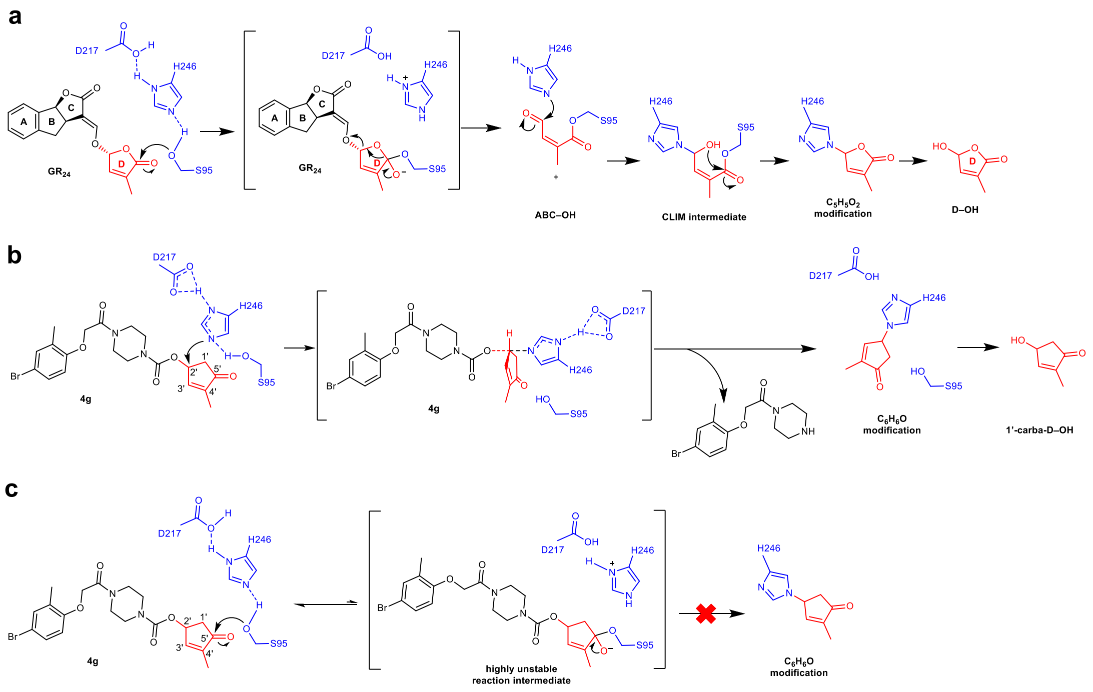

Supplementary Fig. 19. **Proposed schematic diagram of the ShHTL7-mediated agonists hydrolysis pathway. a,** Proposed schematic diagram of the ShHTL7-mediated GR24 hydrolysis pathway in the literatures[11,12]. **b**, Proposed schematic diagram of the ShHTL7-mediated **4g** hydrolysis pathway. The process begins with the nucleophilic attack by NE2 atom of His246 to the 2′C atom of **4g** which, in turn, directly forms C_6H_6O modification of the His246. Similar examples of NE2 atoms of histidines acting as nucleophiles can also be found in the literature[13-22]. **c**, Hydrolysis reaction begins with a nucleophilic attack by Ser95 to 5′ C atom of **4g** cannot form C_6H_6O modification on His246. Because reaction proceeds through this pathway should undergo several processes like ShHTL7-mediated *rac*-GR24 hydrolysis pathway in **a**. If the reaction happens in this way, it should first generate the highly unstable reaction intermediate, followed by electron transfer in the 1′-carba-D ring of the intermediate. Due to the chemical properties of the 1′-carba-D ring, which makes it impossible for electron transfer in the 1′-carba-D ring like the D ring in *rac*-GR24. Therefore, the highly unstable reaction intermediate will immediately transfer to **4g**.

a

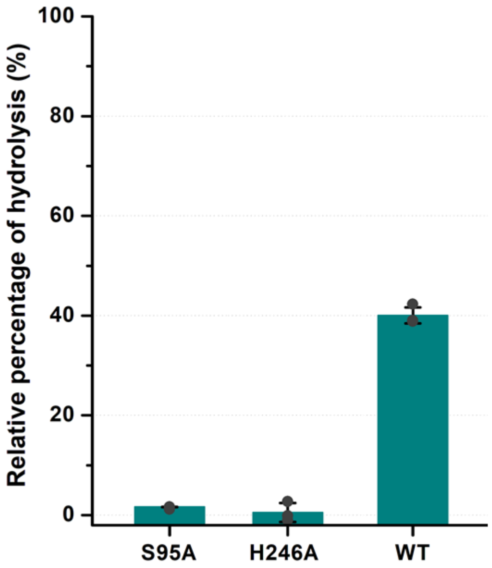

b

Competitive ShHTL7 (mutants) inhibitory activity

	IC_{50} (μM)	
Name	(*R*)-4a	(*S*)-4a
WT	35.80 ± 3.69	0.94 ± 0.11
Y26F	29.94 ± 1.36	0.23 ± 0.14
Y26K	/[a]	/
S95C	43.72 ± 3.65	0.29 ± 0.05
S95A	/	/
T121S	4.90 ± 0.21	0.80 ± 0.25
L124K	1.00 ± 0.10	0.44 ± 0.06
L124M	8.14 ± 0.63	2.58 ± 0.35
F134A	58.11 ± 10.05	0.53 ± 0.11
F134K	62.06 ± 0.45	0.61 ± 0.11
T142L	1.88 ± 0.06	0.30 ± 0.10
T142K	4.13 ± 0.46	0.20 ± 0.12
T157A	2.61 ± 0.17	0.17 ± 0.05
T157Y	23.25 ± 5.92	0.95 ± 0.04
L161M	34.30 ± 10.99	0.67 ± 0.18
L161A	100.20 ± 13.10	0.15 ± 0.08
T190F	4.69 ± 0.66	1.07 ± 0.15
T190G	26.43 ± 0.68	0.57 ± 0.12
M219A	> 100	6.53 ± 1.04
M219V	21.98 ± 1.73	0.35 ± 0.11
M219K	/	/
H246A	/	/

[a]No enzymatic activity.

Supplementary Fig. 20. Effect of key amino acid residues on the hydrolysis of ShHTL7 and binding affinity. a, Relative percentage of hydrolysis of compound (*S*)-**4a** by ShHTL7^{S95A}, ShHTL7^{H246A}, and ShHTL7. The error bar indicates SD (n = 3 biologically independent experiments). **b**, Competitive mutants of ShHTL7 inhibitory activity in YLG assay. Data are the means ± SD (n = 3 biologically independent experiments).

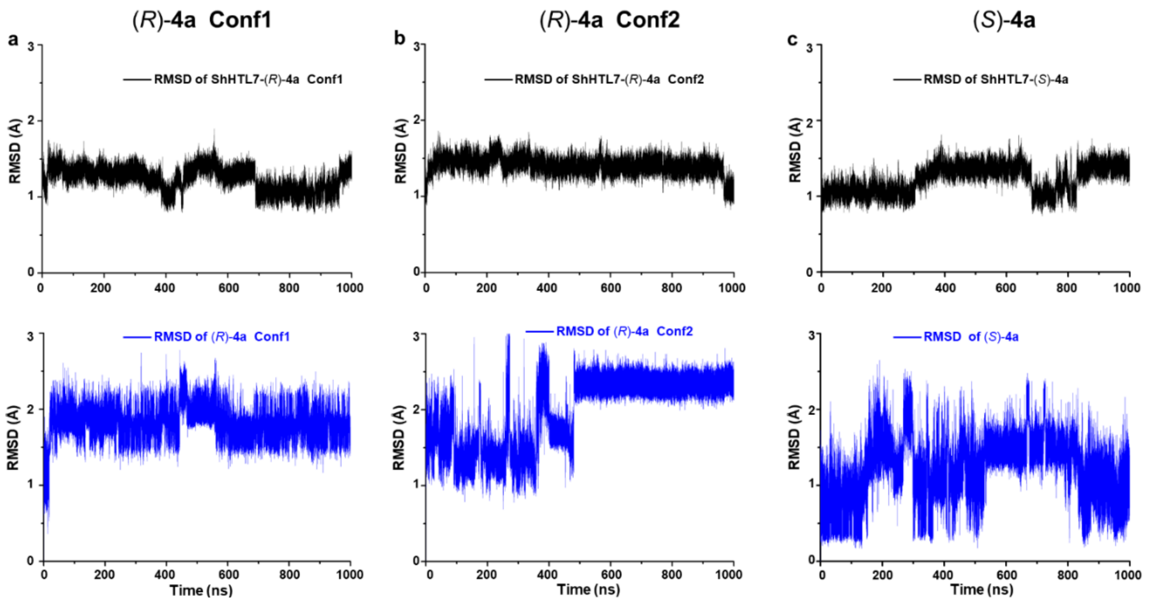

Supplementary Fig. 21. **Root mean square deviation (RMSD) analysis of the N, CA, C atoms of ShHTL7-ligand complexes and the heavy atoms of ligands with respect to the starting structures over the 1000 ns trajectories. a**, RMSD of the N, CA, C atoms of ShHTL7-(*R*)-**4a** (Conf1) and the heavy atoms of (*R*)-**4a** (Conf1) during the MD simulation. **b**, RMSD of the N, CA, C atoms of ShHTL7-(*R*)-**4a** (Conf2) and the heavy atoms of (*R*)-**4a** (Conf2) during the MD simulation. **c**, RMSD of the N, CA, C atoms of ShHTL7-(*S*)-**4a** and the heavy atoms of (*S*)-**4a** during the MD simulation.

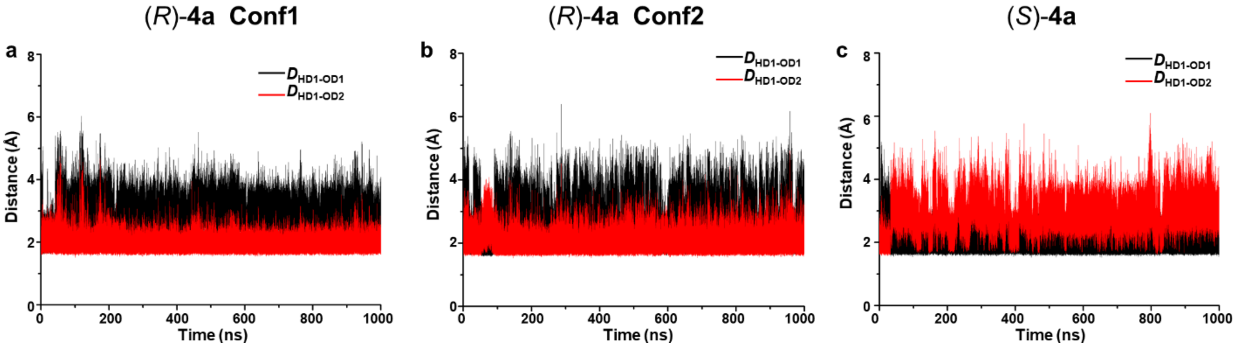

Supplementary Fig. 22. The distances of $D_{HD1-OD1}$ and $D_{HD1-OD2}$ of the ShHTL7-ligand complexes during the 1000 ns simulation. $D_{HD1-OD1}$ is shown in the black line and $D_{HD1-OD2}$ is shown in the red line. **a**, $D_{HD1-OD1}$ and $D_{HD1-OD2}$ of ShHTL7-(*R*)-**4a** (Conf1) complex. **b**, $D_{HD1-OD1}$, and $D_{HD1-OD2}$ of ShHTL7-(*R*)-**4a** (Conf2) complex. **c**, $D_{HD1-OD1}$, and $D_{HD1-OD2}$ of ShHTL7-(*S*)-**4a** complex.

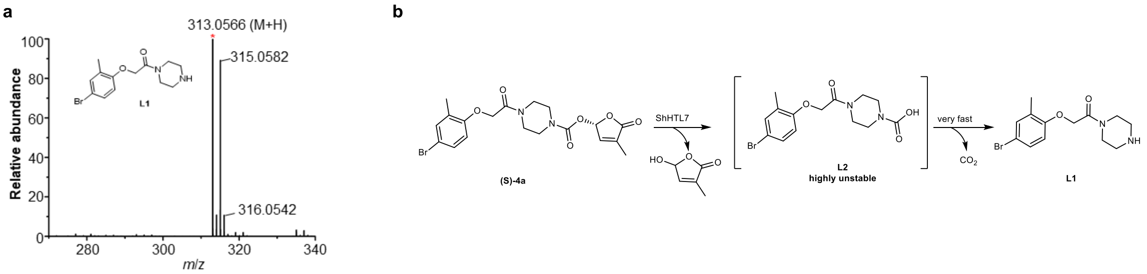

Supplementary Fig. 23. The mass spectrum of the leaving group (compound (*S*)-**4a** without the butenolide ring, designated **L1**) in the (*S*)-**4a** and ShHTL7 reaction system. **b**. A proposed pathway for forming **L1** in the (*S*)-**4a** and ShHTL7 reaction system. After incubating (*S*)-**4a** with ShHTL7, the D-ring was covalently linked to the His246 and released the highly unstable intermediate **L2**. The **L2** would immediately degrade to **L1** by releasing one molecule of CO_2, which, in turn, could promote the enzymatic catalytic reaction.

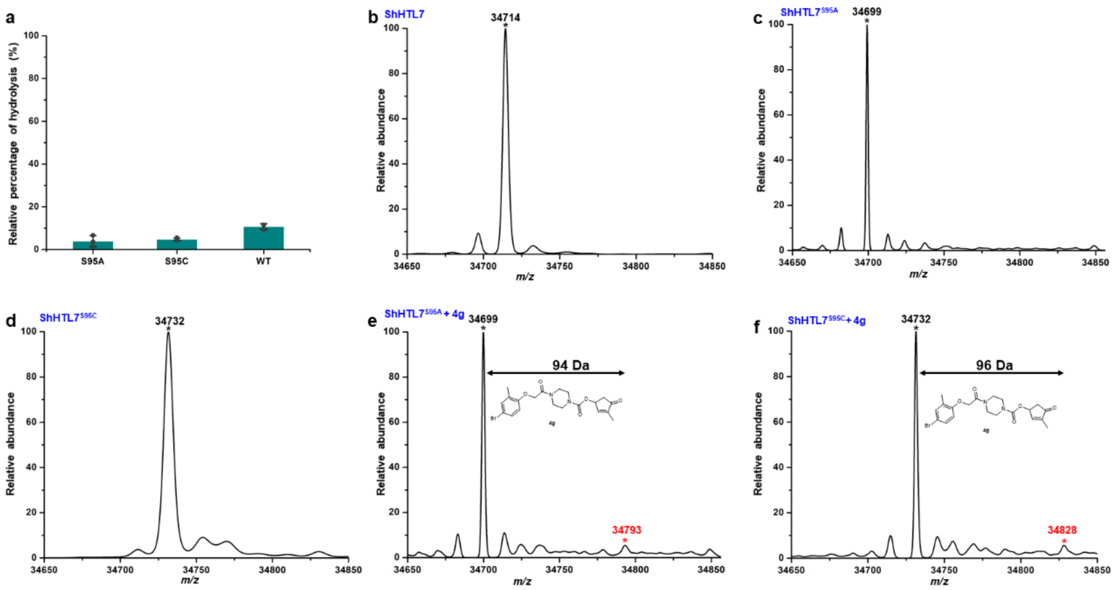

Supplementary Fig. 24. **a**, Relative percentage of hydrolysis of compound **4g** by ShHTL7^{S95A}, ShHTL7^{S95C}, and ShHTL7. The error bar indicates SD (n = 3 biologically independent experiments). **b**, LC-MS analysis of the full-length His-tagged ShHTL7 in native condition. **c**, LC-MS analysis of the full-length His-tagged ShHTL7^{S95A} in native condition, the mass of 34699 Da (reduction of 15 Da compared with that of ShHTL7) is the peak of ShHTL7^{S95A}. **d**, LC-MS analysis of full-length His-tagged ShHTL7^{S95C} in native condition, the mass increment of 18 Da compared with that of ShHTL7 suggesting that ShHTL7^{S95C} is in reduction state. **e**, LC-MS analysis of the **4g**–ShHTL7^{S95A} reaction system in the native condition, an increase of *m*/*z* by 94 Da indicating that the modified D-ring of **4g** is covalently linked to the ShHTL7^{S95A}. **f**, LC-MS analysis of the **4g**–ShHTL7^{S95C} reaction system in the native condition, an increase of *m*/*z* by 96 Da indicating that the modified D-ring of **4g** is covalently linked to the ShHTL7^{S95C}.

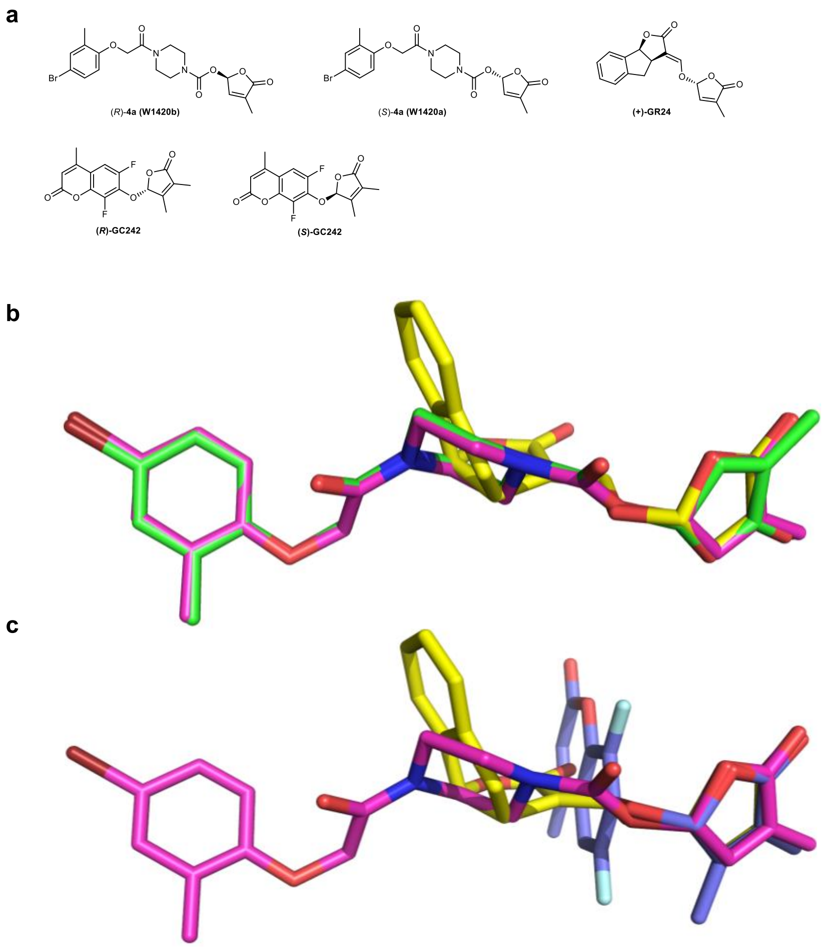

Supplementary Fig. 25. **Alignment of compounds (*R*)-4a**, **(*S*)-4a**, **(+)-GR24, and (*R*)-GC242. a**, chemical structures of (*R*)**-4a**, (*S*)**-4a**, **(+)-**GR24, (*R*)**-GC242,** and (*S*)**-GC242**. **b**, alignment of (*R*)-**4a**, (*S*)**-4a,** and (+)-GR24. (*R*)-**4a** is shown in green stick, (*S*)-**4a** is shown in magenta stick, and (+)-GR24 is shown in the yellow stick. **c**, alignment of (*S*)**-4a**, (+)-GR24, and (*R*)**-GC242**. (*S*)**-4a** is shown in magenta stick, (+)-GR24 is shown in yellow stick, and (*R*)**-GC242** is shown in the blue stick.

	ShHTL1	ShHTL2	ShHTL3	ShHTL4	ShHTL5	ShHTL6	ShHTL7	ShHTL8	ShHTL9	ShHTL10	ShHTL11	PAKAI2c	PAKAI2d1	PAKAI2d2	PAKAI2d3	PAKAI2d4
**ShHTL1**	100	64	64	59	59	63	**59**	59	61	53	53	86	59	57	63	59
**ShHTL2**	64	100	84	66	67	69	**64**	66	68	59	59	69	63	58	66	64
**ShHTL3**	64	84	100	66	66	69	**62**	63	65	57	57	68	62	58	64	62
**ShHTL4**	59	66	66	100	85	74	**72**	71	74	63	62	63	62	62	64	64
**ShHTL5**	59	67	66	85	100	72	**69**	69	70	64	62	62	62	59	64	64
**ShHTL6**	63	69	69	74	72	100	**73**	70	72	63	62	69	68	64	70	70
**ShHTL7**	**59**	**64**	**62**	**72**	**69**	**73**	**100**	**83**	**72**	**59**	**59**	**62**	**63**	**63**	**63**	**62**
**ShHTL8**	59	66	63	71	69	70	**83**	100	70	60	59	62	64	63	64	65
**ShHTL9**	61	68	65	74	70	72	**72**	70	100	61	60	66	64	59	65	65
**ShHTL10**	53	59	57	63	64	63	**59**	60	61	100	85	55	53	54	56	59
**ShHTL11**	53	59	57	62	62	62	**59**	59	60	85	100	54	52	53	56	57
**PAKAI2c**	86	69	68	63	62	69	**62**	62	66	55	54	100	65	59	67	64
**PAKAI2d1**	59	63	62	62	62	68	**63**	64	64	53	52	65	100	61	72	67
**PAKAI2d2**	57	58	58	62	59	64	**63**	63	59	54	53	59	61	100	64	62
**PAKAI2d3**	63	66	64	64	64	70	**63**	64	65	56	56	67	72	64	100	72
**PAKAI2d4**	59	64	62	64	64	70	**62**	65	65	59	57	64	67	62	72	100

Supplementary Fig. 26. Sequence similarity comparison of paKAI2ds and ShHTLs. The multiple sequence alignment was analyzed by https://www.ebi.ac.uk/Tools/msa/muscle/.

	**4a**	**(*R*)-4a**	**(*S*)-4a**	**4g**	**4h**	**4i**	*rac*-GR24
ShHTL2	55.76 ±6.58	66.08 ±3.16	47.42 ±3.02	65.24 ±0.72	76.57 ±3.74	61.94 ±10.13	25.65 ±0.52
ShHTL3	43.03 ±1.58	67.32 ±8.14	15.65 ±1.09	34.07 ±6.03	>100	>100	8.40 ±1.17
ShHTL4	4.95 ±1.18	20.99 ±1.66	2.56 ±0.37	17.71 ±1.59	12.09 ±2.86	17.52 ±3.27	0.25 ±0.02
ShHTL5	15.69 ±2.70	43.91 ±6.97	5.88 ±0.82	39.18 ±3.15	38.11 ±4.74	44.70 ±3.69	6.49 ±0.53
ShHTL6	23.46 ±4.99	>100	8.05 ±1.69	50.63 ±12.21	>100	67.88 ±12.22	2.13 ±0.22
ShHTL7	1.21 ±0.22	35.80 ±3.69	0.94 ±0.11	2.22 ±0.77	4.84 ±0.56	5.18 ±0.81	0.70 ±0.13
ShHTL8	17.15 ±3.79	60.52 ±8.50	8.54 ±3.15	58.27 ±5.60	75.82 ±8.61	73.09 ±2.17	5.81 ±1.17
ShHTL9	22.93 ±3.25	47.31 ±3.17	15.39 ±4.11	38.71 ±7.52	>100	20.06 ±6.44	4.71 ±0.58
ShHTL10	33.93 ±4.96	52.54 ±8.25	12.03 ±0.66	53.14 ±3.09	43.60 ±3.24	64.99 ±6.27	5.67 ±0.83
ShHTL11	10.73 ±2.09	>100	5.33 ±1.40	56.25 ±1.78	>100	93.04 ±7.74	3.19 ±0.07
Phelipanche_aegyptiaca_KAI2c	15.38 ±5.48	51.37 ±6.02	5.57 ±0.43	48.47 ±5.19	32.90 ±8.35	51.74 ±4.59	6.52 ±0.81
Phelipanche_aegyptiaca_KAI2d1	30.47 ±4.94	>100	13.62 ±1.63	24.90 ±1.97	24.88 ±2.90	20.27 ±3.64	2.33 ±0.53
Phelipanche_aegyptiaca_KAI2d2	45.34 ±4.09	>100	37.77 ±3.44	69.98 ±6.72	70.13 ±2.42	56.45 ±1.96	3.83 ±1.17
Phelipanche_aegyptiaca_KAI2d3	28.22 ±2.12	84.90 ±6.50	9.45 ±2.13	49.19 ±11.07	37.91 ±6.32	44.27 ±6.15	1.95 ±0.37
Phelipanche_aegyptiaca_KAI2d4	20.47 ±5.08	>100	6.92 ±1.33	79.56 ±5.00	>100	29.77 ±2.52	6.50 ±0.71

IC_{50} (μM)

Supplementary Fig. 27. Binding of compounds **4a**, (*R*)- and (*S*)-**4a**, and **4g-i** to the HTL proteins in *S. hermonthica* and KAI2 protein homologs in *P. aegyptiaca*. In the competitive inhibitory activity assay, YLG (3 μM) was used as the fluorogenic substrate. Error bar indicates SD (n = 3 biologically independent experiments).

Supplementary Table 1. Structures, *P. aegyptiaca* seed germination activity, and ShHTL7 inhibitory activity of compounds **1-3**.

compds	structure	*P. aegyptiaca germination*(%)[a]	IC_{50}(μM)[b]	compds	structure	*P. aegyptiaca germination*(%)[a]	IC_{50}(μM)[b]
**1**	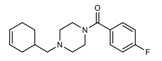	39.4±10.6	15.87±1.47	**3i**	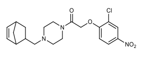	33.5±1.0	11.56±0.61
**2f**	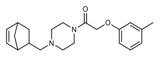	20.5±2.5	10.67±0.96	**3j**	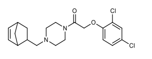	54.7±3.6	9.22±0.35
**2h**	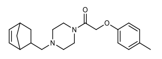	27.4±10.1	22.03±4.97	**3k**	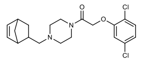	43.5±2.5	32.22±1.99
**3a**	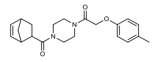	19.4±2.6	35.57±3.84	**3l**	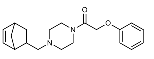	26.3±0.7	18.00±1.32
**3b**	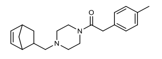	8.9±5.4	23.17±1.59	**3m**	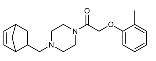	29.7±2.4	9.16±1.20
**3c**	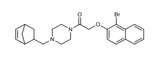	17.1±0.9	6.12±0.34	**3n**	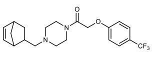	47.8±3.6	12.78±2.97
**3d**	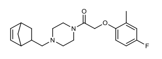	23.1±0.3	10.71±2.93	**3o**	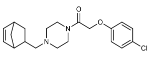	21.4±3.6	22.00±2.43
**3e**	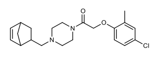	7.0±1.0	8.28±1.12	**3p**	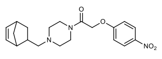	18.6±2.7	15.59±0.37
**3f**	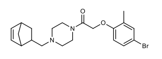	8.3±3.0	7.45±1.03	**3q**	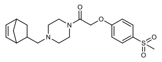	34.8±1.7	24.09±6.49
(2*R*)-**3f**	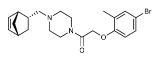	15.1±2.5	5.67±0.46	**3r**	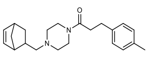	34.2±6.9	34.19±6.90
(2*S*)-**3f**	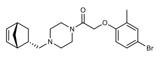	6.7±3.4	6.40±1.25	ZJT1	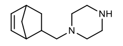	1.6±2.3	/[c]
**3g**	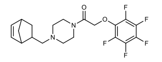	5.7±1.9	0.23±0.03	*rac*-GR24		96.4±2.6	0.73±0.11
(2*R*)-**3g**	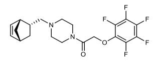	9.1±0.4	0.13±0.01	0.1%Ac		6.2±2.9	
(2*S*)-**3g**	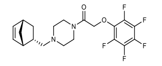	9.6±4.6	2.04±0.06				
**3h**	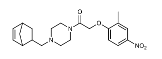	22.0±2.0	10.03±0.59				

[a]Compounds were tested at 10 μM. [b]YLG was used as the substrate in the IC_{50} assay. [c]means no activity. Data are the means ± SD (n = 3 biologically independent experiments).

Supplementary Table 2. X-ray diffraction data collection and refinement statistics of ShHTL7.

**Crystal**	**ShHTL7**
Diffraction source	BL-19U1, SSRF
Wavelength (Å)	0.9800
Space group	C2
Unit-cell parameters (Å, °)	*a*=92.548, *b*=92.500, *c* =75.428<br>*α*=*γ*=90.00, *β*=116.92
Resolution range (Å)	50.0-2.30
No. of unique reflections	25025
Completeness (%)	99.6(99.5)
Multiplicity	3.3(3.2)
$< I/\sigma (I)>$	13.77(2.04)
R_{meas} (%)	11.8(46.2)
Refinement	
R_{factor} (%)	18.34
R_{free} (%)	23.52
Number of protein atoms	4216
Number of ligands/ion	0
Number of water molecules	65
Average B-factor (Å^2)	56.40
RMSD from ideal values	
Bond length (Å)	0.009
Bond angle (°)	1.165
Residues in (%)	
Most favored	92.8
Generously allowed	6.8
Disallowed	0.4
Protein Data Bank entry	6A9D

Supplementary Table 3. Binding features of the Ser and His in the catalytic trial of SL receptors related α/β-hydrolase proteins.

**Protein**[a]	**H-bond partner**[b]	**Distance (Å)**[c]	**Ref.**
AtD14-4ih4	S97-H247	2.9	[23]
AtD14L-3w06	S95-H246	3.0	[24]
OsD14-4iha	S97-H247	2.5	[23]
AtKAI-4jym	S95-H246	1.8	[25]
AtKAI-4jyp	S95-H246	1.8	[25]
ShHTL1-5z7w	S95-H246	1.6	[26]
ShHTL4-5z7x	S95-H246	2.9	[26]
ShHTL5-5cbk	S95-H246	3.5	[27]
ShHTL7-5z7y	S95-H246	2.1	[26]
ShHTL7-5z82	S95-H246	2.1	[28]
ShHTL7-6a9d	S95-H246	2.1	[d]
ShHTL8-6J2R	S96-H247	2.4	[29]
ShKAI2L-5dnu	S95-H246	3.3	[30]
ShKAI2L-5dnv	S95-H246	2.2	[30]
ShKAI2L-5dnw	S95-H246	1.9	[30]
KAI2_ply2-5z9h	S95-H246	1.7	[31]
KAI2-4hrx	S95-H246	1.8	[32]
Pp-KAI2-like E-6azc	S97-H248	1.6	[33]
PpKAI2-like H-6azd	S98-H249	1.8	[33]

[a]Name and PDB entry for the α/β-hydrolase protein structures. [b]Hydrogen bond donors and acceptors. [c]The distances of hydrogen bonds between the HG atoms of Ser and NE2 atoms of His. To make the data consistent with our MD calculation, the hydrogen atoms of the PDB structures were added by the pdb4amber, a program in amber14. [d]The current work.

Supplementary Table 4. The hydrogen analysis of the (S)-4a–ShHTL7 system in the1000 ns of the MD.

#Acceptor	DonorH	Donor	AvgDist/Å	AvgAng/°
RES_269@O2	THR_157@HG1	THR_157@OG1	2.90	160.30
RES_269@O3	TYR_174@HH	TYR_174@OH	3.01	143.36
RES_269@O5	MET_96@H	MET_96@N	3.25	135.70
RES_269@O5	TYR_26@H	TYR_26@N	3.28	144.76

Supplementary Table 5. The nucleophilic substitution reaction conditions of As-his-ome to the 2′C atom of 4g.[a]

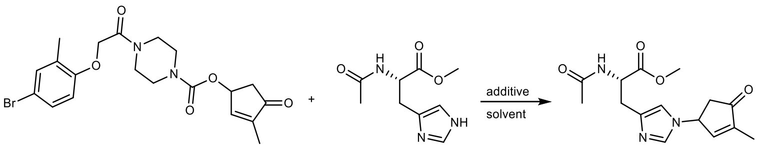

entry	additive (equiv)	solvent	time (h)	temperature (°C)	yield (%)[b]
1	NaH (1.2)	THF	6	0 to 25	trace
2	K_2CO_3 (1.25)	DMF	6	25	trace
3	DBU (1.5)	THF	6	25	trace

[a]Reactions were performed on a 0.033 mmol scale of **4g** and 10 mL of solvent under the given conditions. [b]The yields were determined by UPLC-HRMS.

Supplementary Table 6. List of primers used for constructing yeast expression vectors.

Primers	Sequences
BD-	Forward: CGCGGATCCGTATGAGTCAACACAACATCTTAG
AtD14	Reverse: ACGCGTCGACTCACCGAGGAAGAGCTCGCCGGAG
AD-	Forward: ACGTACCAGATTACGCTCATATGATGTCTGCGAAGAAGATTGTGTTG
AtMAX2	Reverse: GGATCCATCGAGCTCGAGCTGCATCAGTCAATGATGTTGCGGCTG
AD-	Forward: GGGAATTCCATATGATGAGAGCTGGTCTGTCCACTATTC
SMAX1	Reverse: ACGCGTCGACTCATACTGCCAAAGTAATAG
AD-	Forward: AGAAGAGAAAGGTGGCGGCCGCAATGTCTGCGAAGAAGATTGTGTTG
ShMAX2	Reverse: AGCCCGAAGATCTTCGGGCTAATTAATCGGAGATCTGGCGACGG
BD-	Forward: TCTCAGAGGAGGACCTGCATATGATGAGCAGCATTGGCCTGGCG
ShHTL7	Reverse: TGCGGCCGCTGCAGGTCGACGGATCCTCAGTGATCGGTAATATCCTG

Supplementary Table 7. Properties and K_D values of compounds 4, SPL7, and *rac*-GR24.

compds	Structure	Acceptor_Count[a]	CLogP[a]	Polar_Surface_Area[a]	K_D/μM[b]
**4a**	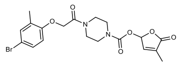	8	3.33	100.66	2.07±0.51
***R*-4a**	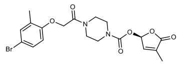	8	3.33	102.26	1.69±0.04
***S*-4a**	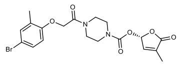	8	3.33	100.25	0.79±0.06
**4b**	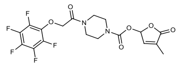	8	2.29	109.40	2.21±0.42
**4c**	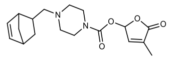	6	2.94	93.79	13.89±2.6
**4d**	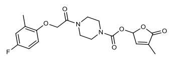	8	2.61	100.12	0.82±0.26
**4e**	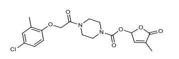	8	3.18	100.43	6.33±3.1
**4f**	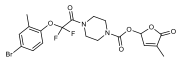	8	5.08	98.75	1.96±0.29
**4g**	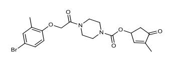	7	3.76	72.44	34.77±3.90
**4h**	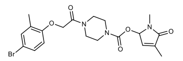	8	3.64	91.82	12.09±0.94
**4i**	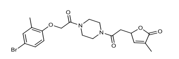	7	2.84	101.49	2.93±0.087
**4j**	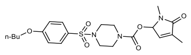	9	3.81	103.82	18.10±6.29
**SPL7**	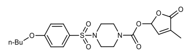	9	3.49	141.94	4.81±0.02
***rac*-GR24**		5	0.61	95.42	0.93±0.17

[a]Properties were calculated by Sybyl 6.9. [b]The binding affinities between ShHTL7 and agonists were obtained by SPR assay.

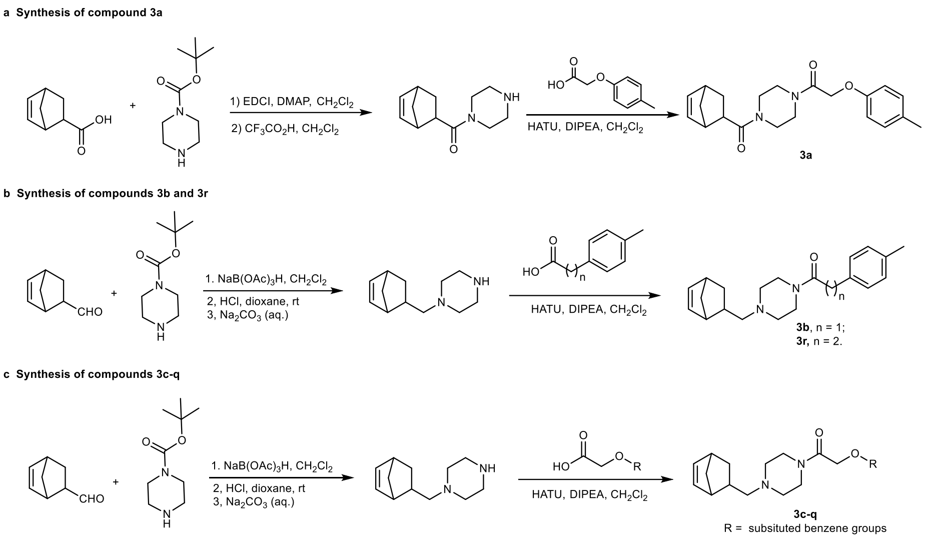

Supplementary Scheme 1. The synthetic routes of compounds 3a-r.

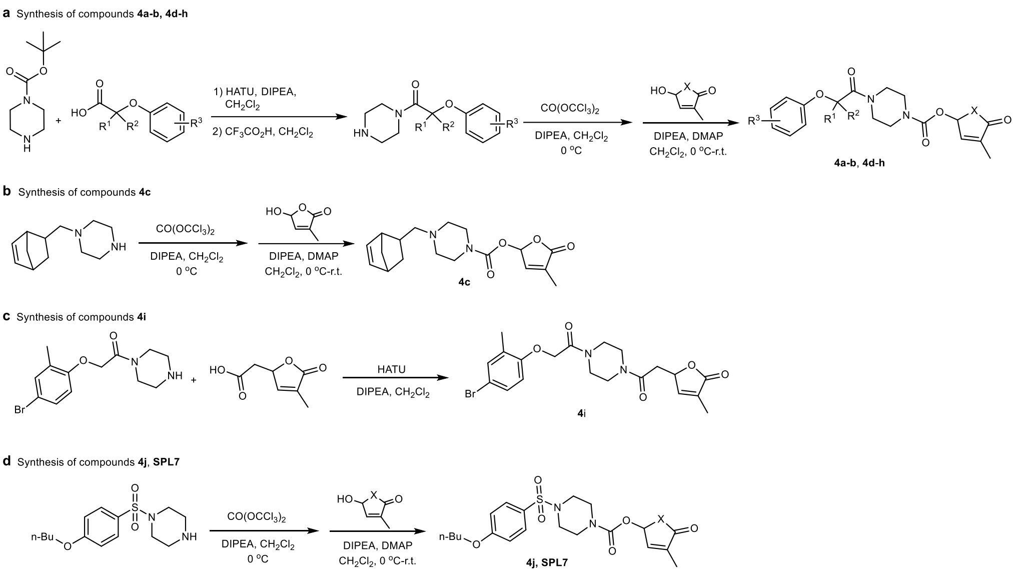

Supplementary Scheme 2. The synthetic routes of compounds 4a-j and SPL7.

Supplementary Note. Synthesis of compound **3a**.

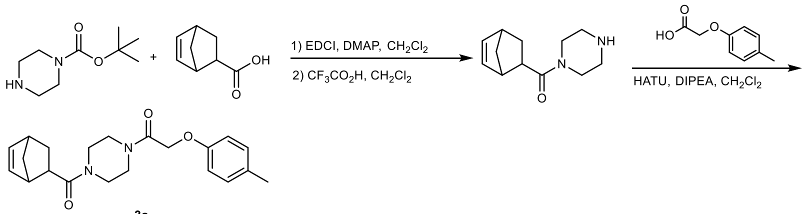

1-(3-dimethylaminopropyl)-3-ethylcarbodiimide hydrochloride (EDCI) (27.1 g, 141.3 mmol) was added to a solution of bicyclo[2.2.1]hept-5-ene-2-carboxylic acid (mixture of endo and exo) (13 g, 94.2 mmol) in CH_2Cl_2 (100 mL) with stirring at 0 °C. After 15 min, *tert*-butyl piperazine-1-carboxylate (18.4 g, 98.9 mmol) and 4-dimethylaminopyridine (DMAP) (12.6 g, 103.5 mmol) were added successively to the solution. The reaction mixture was stirred at room temperature for 12 hours, then H_2O (100 mL) was added to the mixture and stirred vigorously for 10 min. The organic layer was separated and washed by saturated Na_2CO_3 aqueous solution (100 mL), and 1 mol/L HCl (100 mL), dried over $MgSO_4$ and filtered. The filtrate was used directly in the next step without purification. CF_3COOH (20 mL) was added drop-wise to the solution; after stirring at room temperature for 2 h, the solvent of the reaction was removed under reduced temperature. The resulting residue was dissolved in CH_2Cl_2 (150 mL), and saturated Na_2CO_3 aqueous solution (200 mL) was added slowly to the solution. The mixture was stirred vigorously for 30 min. The organic layer was separated, concentrated by rotary evaporation, and purified by column chromatography ($V_{\text{petroleum ether}}$: V_{acetone} : V_{CH2Cl2} : V_{Et3N}= 20 : 5 : 5 : 1) to give the desired compound (13.78 g, yield 71%).

N-ethyl-*N*-isopropylpropan-2-amine (DIPEA) (0.34 g, 2.6 mmol) was added to a mixture of 2-(*p*-tolyloxy)acetic acid (0.24 g, 1.46 mmol), 2-(7-azabenzotriazol-1-yl)-*N,N,N',N'*-tetramethyluronium hexafluorophosphate (HATU) (0.72 g 1.9 mmol), and bicyclo[2.2.1]hept-5-en-2-yl(piperazin-1-yl)methanone (0.3 g, 1.46 mmol) in CH_2Cl_2 (50 mL) with stirring. The mixture was stirred at room temperature for 6 h. H_2O (30 mL) was added, and the mixture was stirred vigorously

for 10 min. The organic layer was separated, and washed by saturated Na_2CO_3 aqueous solution (30 mL) and 1 mol/L HCl (30 mL), concentrated to dryness. The residue was first purified by flash chromatography on silica gel ($V_{petroleum\ ether}$: $V_{acetone}$ = 3 : 1) and then recrystallized from diethyl ether to afford to give pure compound **3a** (0.44 g, yield 85%). ^{1}H NMR (400 MHz, $CDCl_3$) δ 7.09 (m, 2H), 6.88 – 6.79 (m, 2H), 6.22-6.15 (m, 1H), 6.15-6.10 (m, 1H), 4.69 (s, 2H), 3.73-3.40 (m, 8H), 2.96 (s, 1H), 2.92 (s, 1H), 2.33-2.24 (m, 4H), 1.87 – 1.78 (m, 1H), 1.59 (d, J = 8.4 Hz, 1H), 1.49 – 1.35 (m, 2H). ^{13}C NMR (101 MHz, $CDCl_3$) δ 174.45, 166.95, 155.40, 138.36, 136.68, 135.71, 132.81, 131.09, 130.07, 114.23, 68.10, 67.79, 47.03, 45.65, 45.41, 45.29, 44.95, 42.08, 41.86, 41.52, 41.04, 31.27, 20.41. HRMS (ESI): calcd for $C_{21}H_{26}N_2NaO_3$ $[M+Na]^+$: 377.1841, found: 377.1839.

Supplementary Note. Synthesis of compound **3b**.

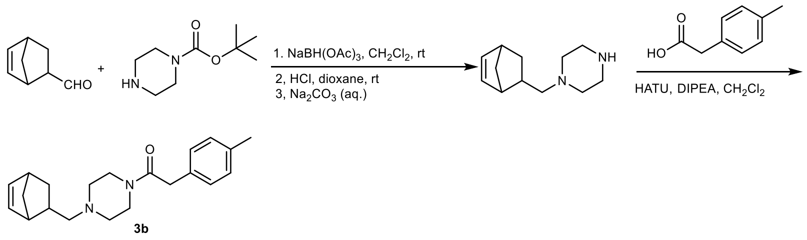

NaBH(OAc)3 (14.45 g, 68.2 mmol) was added slowly to a solution of bicyclo[2.2.1]hept-5-ene-2-carbaldehyde (mixture of endo and exo) (5.55 g, 45.5 mmol) and *tert*-butyl piperazine-1-carboxylate (11 g, 59.15 mmol) in CH_2Cl_2 (150 mL) at 0 ºC. After stirring under argon atmosphere for 30 min, the reaction mixture was moved to room temperature and stirred for another 12 h. After completing the reaction check by TLC ($V_{petroleum\ ether}$:$V_{acetone}$: V_{Et3N}= 20:5:1) detection. Ice cold water (100 mL) was added dropwise to the reaction mixture to quench the reaction. After stirring vigorously for 15 min, the organic layer was separated and washed with saturated NaCl solution (100 mL) and dried over anhydrous Na_2SO_4. The organic solvent was removed by rotary evaporation, and the residue was dissolved in HCl-dioxane (4 mol/L, 136 mL) solution and stirred at room temperature for 6 h. Upon

completing the reaction, the organic solvent was removed under reduced pressure. Diethyl ether (150 mL) was added to the flask and stirred vigorously for 15 min. The resulting solid was filtered and washed with diethyl ether (100 mL); without further purification, the solid was transferred into a flask. CH_2Cl_2 (150 mL) and saturated Na_2CO_3 aqueous solution (100 mL) were added carefully to the flask. After stirring vigorously for 30 min, the organic layer was separated, and the aqueous layer was rewashed by CH_2Cl_2 (50 mL). The combined organic layer was washed with saturated NaCl solution (100 mL) and concentrated to dryness. The crude compound was purified by flash chromatography on silica gel ($V_{\text{petroleum ether}}$: V_{acetone} : V_{CH2Cl2} : V_{Et3N}= 20 : 5 : 5 : 1) to give the desired compound (7.51g, yield 86%).

To a solution of 2-(*p*-tolyl)acetic acid (0.2 g, 1.33 mmol), HATU (0.66 g 1.73 mmol), and 1-(bicyclo[2.2.1]hept-5-en-2-ylmethyl)piperazine (0.26 g, 1.33 mmol) in CH_2Cl_2 (50 mL) was added DIPEA (0.34 g, 2.6 mmol) at room temperature. After stirring at room temperature for 6 h, H_2O (30 mL) was added to the mixture. The organic layer was separated, the water layer was washed again with CH_2Cl_2 (20 mL). The combined organic layer was washed with saturated aqueous NaCl solution (30 mL) and concentrated to dryness. The crude compound was purified by flash chromatography on silica gel ($V_{\text{petroleum ether}}$:$\text{V}_{\text{acetone}}$: V_{Et3N}= 20:5:1) and then recrystallized from diethyl ether to afford to give pure compound **3b** (0.29 g, yield 67%). ^{1}H NMR (400 MHz, $CDCl_3$) δ 7.12 (s, 4H), 6.16 – 5.86 (m, 2H), 3.68 (s, 2H), 3.64 (s, 2H), 3.44 (s, 2H), 2.86 – 2.74 (m, 2H), 2.45 – 1.78 (m, 11H), 1.39 (dd, *J* = 8.0, 1.6 Hz, 1H), 1.21 (d, *J* = 8.0 Hz, 1H), 0.60 – 0.47 (m, 1H). ^{13}C NMR (101 MHz, $CDCl_3$) δ 169.47, 136.99, 136.65, 136.47, 136.13, 132.35, 131.91, 129.26, 128.32, 64.34, 62.90, 53.37, 53.00, 52.95, 49.32, 46.01, 45.14, 44.97, 44.90, 42.32, 41.72, 41.66, 40.48, 36.11, 36.00, 31.72, 31.34, 20.95. HRMS (ESI): calcd for $C_{21}H_{29}N_2O$ $[M+H]^+$: 325.2280, found: 325.2276.

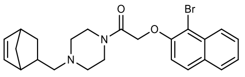

Compound **3c** was synthesized by using the similar methods as **3b**. Yield, 96%. ^{1}H NMR (400 MHz, $CDCl_3$) δ 8.22 (d, J = 8.8 Hz, 1H), 7.83 – 7.76 (m, 2H), 7.58 (t, J = 7.2 Hz, 1H), 7.42 (t, J = 7.2 Hz, 1H), 7.33 (d, J = 8.8 Hz, 1H), 6.18 – 5.88 (m, 2H), 4.91 (s, 2H), 3.75 (s, 2H), 3.65 (s, 2H), 2.82 (s, 1H), 2.77 (s, 1H), 2.59 – 2.31 (m, 4H), 2.31 – 2.20 (m, 1H), 2.19 – 2.09 (m, 1H), 2.07-1.93 (m, 1H), 1.88-1.79 (m, 1H), 1.39 (d, J = 8.0 Hz, 1H), 1.20 (d, J = 8.0 Hz, 1H), 0.54 (d, J = 11.6 Hz, 1H). ^{13}C NMR (101 MHz, $CDCl_3$) δ 165.81, 152.11, 137.06, 136.70, 136.51, 133.00, 132.38, 130.16, 129.09, 128.05, 127.78, 126.17, 124.67, 114.47, 109.13, 69.23, 64.34, 62.88, 53.66, 52.93, 49.36, 45.50, 45.18, 45.01, 44.94, 42.35, 42.17, 41.76, 36.10, 36.02, 31.74, 31.36. HRMS (ESI): calcd for $C_{24}H_{28}BrN_2O_2$ $[M+H]^+$: 455.1334, found: 455.1330.

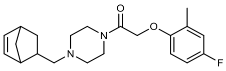

Compound **3d** was synthesized by using the similar methods as **3b**. Yield, 85%; ^{1}H NMR (400 MHz, $CDCl_3$) δ 6.91 – 6.73 (m, 3H), 6.16 – 5.88 (m, 2H), 4.66 (s, 2H), 3.63 (s, 2H), 3.59 (s, 2H), 2.84 (s, 1H), 2.78 (m, 1H), 2.55-2.31 (m 2H), 2.38 (d, J = 13.7 Hz, 2H), 2.28-2.21 (m, 4H), 2.18-2.06 (m, 1H), 2.05-1.94 (m, 1H), 1.92 – 1.80 (m, 1H), 1.41 (dd, J = 8.8, 2.0 Hz, 1H), 1.23 (d, J = 8.4 Hz, 1H), 0.56 (d, J = 11.6 Hz, 1H). ^{13}C NMR (101 MHz, $CDCl_3$) δ 166.23, 157.22 (d, J = 239.6 Hz), 152.08, 137.11, 136.74, 136.50, 132.36, 128.56 (d, J = 7.7 Hz), 117.54 (d, J = 23.0 Hz), 112.50 (d, J = 22.9 Hz), 111.99 (d, J = 8.7 Hz), 68.34, 64.40, 62.96, 53.66, 52.98, 49.38, 45.31, 45.18, 45.02, 44.95, 42.38, 42.06, 41.78, 36.17, 36.08, 31.75, 31.36. HRMS (ESI): calcd for $C_{21}H_{28}FN_2O_2$ $[M+H]^+$: 359.2135, found: 359.2133.

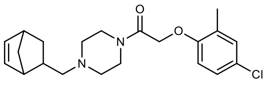

Compound **3e** was synthesized by using the similar methods as **3b**. Yield, 89%. ^{1}H NMR (400 MHz,

CDCl3) δ 7.12 (d, J = 2.0 Hz, 1H), 7.09 (dd, J = 8.4, 2.8 Hz, 1H), 6.76 (d, J = 8.4 Hz, 1H), 6.16 – 5.86 (m, 2H), 4.67 (s, 2H), 3.63 (s, 2H), 3.59 (s, 2H), 2.84 (s, 1H), 2.78 (s, 1H), 2.51 – 2.30 (m, 4H), 2.30 – 2.19 (m, 4H), 2.18 – 2.08 (m, 1H), 1.99 (dd, J = 11.6, 6.8 Hz, 1H), 1.91 – 1.78 (m, 1H), 1.45-1.37 (m, 1H), 1.23 (d, J = 8.0 Hz, 1H), 0.61-51 (m, 1H). ^{13}C NMR (101 MHz, CDCl3) δ 166.02, 154.56, 137.12, 136.75, 136.51, 132.37, 130.61, 128.50, 126.46, 125.89, 112.11, 67.92, 64.39, 62.95, 53.64, 52.95, 49.38, 45.31, 45.19, 45.01, 44.93, 42.38, 42.08, 41.78, 36.14, 36.06, 31.75, 31.36, 16.14. HRMS (ESI): calcd for $C_{21}H_{28}ClN_2O_2$ $[M+H]^+$: 375.1839, found: 375.1835.

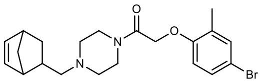

Compound **3f** was synthesized by using the similar methods as **3b**. ^{1}H NMR (400 MHz, CDCl3) δ 7.27 (s, 1H), 7.23 (dd, J = 8.4, 2.4 Hz, 1H), 6.72 (d, J = 8.4 Hz, 1H), 6.21 – 5.89 (m, 2H), 4.67 (s, 2H), 3.64 (brs, 2H), 3.59 (brs, 2H), 2.84 (brs, 1H), 2.78 (brs, 1H), 2.51-2.33 (m, 4H), 2.31 – 2.18 (m, 4H), 2.18-2.10 (m, 1H), 2.06 – 1.96 (m, 1H), 1.90 – 1.79 (m, 1H), 1.44-1.38 (m, 1H), 1.27 – 1.19 (m, 1H), 0.60 – 0.48 (m, 1H). ^{13}C NMR (101 MHz, CDCl3) δ 165.93, 155.06, 137.07, 136.71, 136.49, 133.41, 132.35, 129.45, 128.95, 113.32, 112.59, 67.80, 64.35, 62.92, 53.62, 52.93, 49.36, 45.31, 45.16, 44.98, 44.90, 42.35, 42.07, 41.76, 36.13, 36.04, 31.71, 31.31, 16.05. HRMS (ESI): calcd for $C_{21}H_{28}BrN_2O_2$ $[M+H]^+$: 419.1334, found: 419.1330.

Supplementary Note. Separation of the enantiomers of **3f**.

The chiral isomers of **3f** were analyzed by the Shimadzu LC-20AD CP-HPLC-08 instrument with a CHIRALPAK IF (IF00CD-TB009) 0.46 cm I.D. × 25 cm L chiral column. Parameters set for chiral HPLC used to analyze the isomers were as follows: temperature: 35 °C; wavelength: UV 220 nm; mobile phase: V_{Hexane}/V_{EtOH} = 95/5; injection volume: 5 μL. Two isomers were found in **3f**; the

retention times for isomer **a** ((2*R*)-**3f**), and isomer **b** ((2*S*)**-3f**) were 19.5 and 21.8 min, respectively. The separation of two isomers was performed on the YMC-1 (K-PREP-K-100) preparative HPLC instrument with CHIRALPAK IF 50 ×250 mm, 5 µm column. The parameters set for preparative HPLC were as follows: temperature: 35 °C; detection wavelength: UV 214 nm; mobile phase: V_{Hexane}/V_{EtOH} = 95/5; flow rate: 60 mL/min; injection volume: 3 mL. In the separation process, each peak was collected independently, and the combined solvent of each peak was removed by rotary evaporation at 35 °C. The final purity of (2*R*)-**3f** and (2*S*)**-3f** was checked using analytical HPLC.

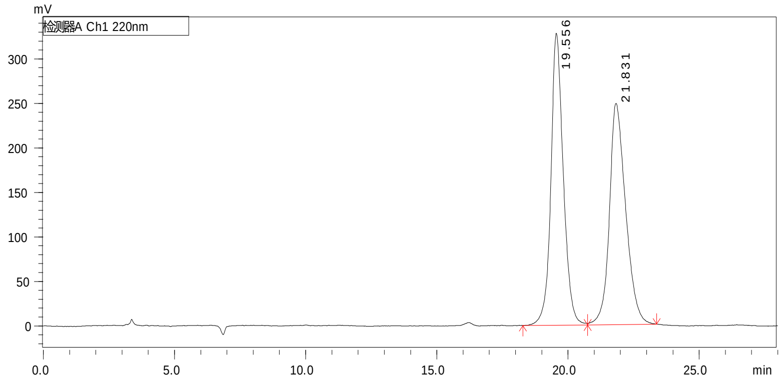

Analysis of **3f** by analytical HPLC.

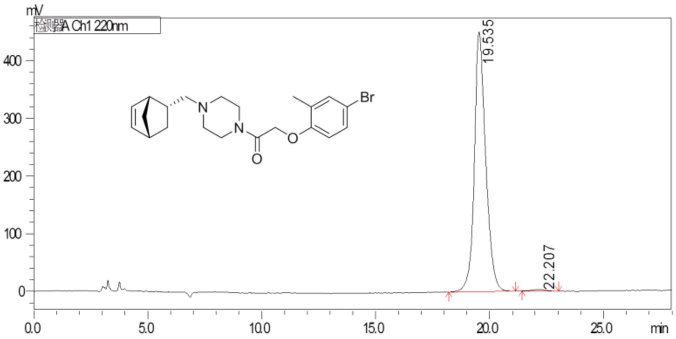

Peak#	Ret. Time	Area	Area%	T.Plate#	Tailing F.	Resolution
1	19.535	15612978	99.258	7897	1.177	--
2	22.207	116781	0.742	13263	0.954	3.237

HPLC analysis of (2*R*)-**3f**

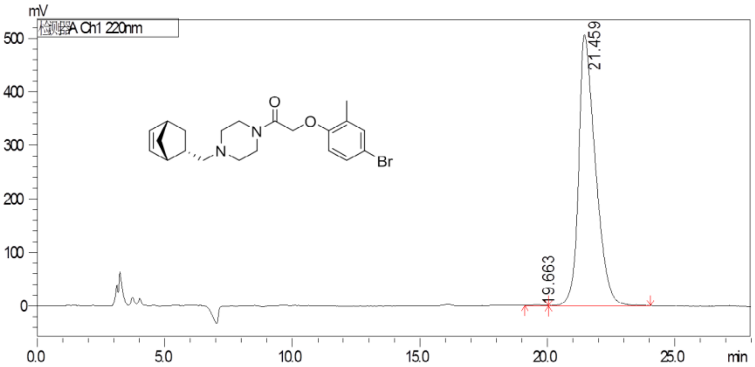

Peak#	Ret. Time	Area	Area%	T.Plate#	Tailing F.	Resolution
1	19.663	43935	0.189	21621	0.880	--
2	21.459	23213081	99.811	5031	1.403	2.058

HPLC analysis of (2*S*)-**3f**

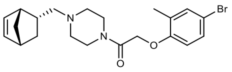

The ^{1}H and ^{13}C NMR spectrum data of (2*R*)-**3f** were identical to **3f**. (2*R*)-**3f** was obtained in a purity of 99.3% (retention time 19.5 min), 98.5% ee. $[\alpha]_D^{20}$: 48.0 (*c* 0.8 in $CHCl_3$). HRMS (ESI): calcd for $C_{21}H_{28}BrN_2O_2$ $[M+H]^+$: 419.1334, found: 419.1330. The single crystals of (2*R*)-**3f** were obtained by slow evaporation from acetone solution. The supplementary crystallographic data for (2*R*)-**3f** has been deposited in the Cambridge Crystallographic Data Centre (CCDC, http://www.ccdc.cam.ac.uk/) under deposition number 1856748.

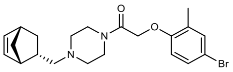

The ^{1}H and ^{13}C NMR spectrum data of (2*S*)-**3f** were identical to **3f**. (2*S*)-**3f** was obtained in a purity of 99.8% (retention time 21.5 min), 99.6 % ee. $[\alpha]_D^{20}$: 48.8 (*c* 0.5 in $CHCl_3$). HRMS (ESI): calcd for $C_{21}H_{28}BrN_2O_2$ $[M+H]^+$: 419.1334, found: 419.1330. The single crystals of (2*S*)-**3f** were obtained by slow evaporation from acetone solution. The supplementary crystallographic data for (2*S*)-**3f** has been deposited in the Cambridge Crystallographic Data Centre (CCDC, http://www.ccdc.cam.ac.uk/) under deposition number 1856749.

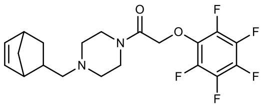

Compound **3g** was synthesized by using the similar methods as **3b**. Yield, 92%, ^{1}H NMR (400 MHz,

$CDCl_3$) δ 6.17 – 5.88 (m, 2H), 4.87 (s, 2H), 3.62 (s, 2H), 3.47 (d, J = 4.0 Hz, 2H), 2.85 (s, 1H), 2.79 (s, 1H), 2.57 – 2.33 (m, 4H), 32-2.21 (m, 1H), 2.21-2.09 (m, 1H), 2.7-1.96 (m, 1H), 1.92-1.78 (m, 1H), 1.47-1.38 (m 1H), 1.24 (d, J = 8.0 Hz, 1H), 0.63-0.50 (m, 1H). ^{13}C NMR (101 MHz, $CDCl_3$) δ 164.73, 142.62 (m, 1C), 140.12 (m, 1C), 139.19 (m, 1C), 137.41 (dt, J = 15.6 Hz, J = 255.2 Hz, 1C), 136.76, 136.65 (m, 1C), 132.81 (m, 1C), 71.21, 71.18, 71.14, 64.37, 62.94, 53.35, 52.80, 49.38, 45.19, 45.00, 44.92, 42.39, 41.95, 41.79, 36.16, 36.08, 31.73, 31.33. HRMS (ESI): calcd for $C_{20}H_{22}F_5N_2O_2$ $[M+H]^+$: 417.1601, found: 417.1598.

Supplementary Note. Separation of the enantiomers of **3g**.

The chiral isomers of **3g** were analyzed by the Shimadzu LC-20AD CP-HPLC-08 instrument with a CHIRALPAK IF (IF00CD-TB009) 0.46 cm I.D. × 25 cm L chiral column. Parameters set for chiral HPLC used for the analysis of the isomers were as follows: temperature: 35 °C; wavelength: UV 220 nm; mobile phase: $V_{\text{Hexane}}/V_{\text{EtOH}}$ = 95/5; injection volume: 5 μL. Four isomers were found in **3g**; the retention times for isomer a was 27.3 min (minor), isomer b was 31.0 min (major), isomer c was 36.5 min (minor), and isomer d was 39.3 min (major). The separation of isomers was performed on the YMC-1 (K-PREP-K-100) preparative HPLC instrument with CHIRALPAK IF 50 ×250 mm, 5 μm column. The parameters set for preparative HPLC were as follows: temperature: 35 °C; detection wavelength: UV 214 nm; mobile phase: $V_{\text{Hexane}}/V_{\text{EtOH}}$ = 95/5; flow rate: 60 mL/min; injection volume: 3 mL. Three rounds of separation were performed to separate the two major isomers (b and d): firstly, the former two peaks (isomers a and b**)** and **the** latter two peaks (isomers c and d) as collected; secondly, the separation of isomers a and b, and collected the peak of isomer b; thirdly, separation of isomers c and d, collected the peak of isomer d. The solvent of isomers b and d was removed by rotary evaporation at 35 ^{o}C. The final purity of (2*R*)-**3g** and (2*S*)-**3g** was checked by analytical HPLC.

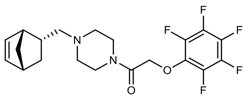

The ^{1}H and ^{13}C NMR spectrum data of (2*R*)-**3g** were identical to **3g**. (2*R*)-**3g** was obtained in a purity of 98.8% (retention time 31.6 min), 97.6% ee. $[\alpha]_D^{20}$: 42.5 (*c* 0.8 in $CHCl_3$). HRMS (ESI): calcd for $C_{20}H_{22}F_5N_2O_2$ $[M+H]^+$: 417.1601, found: 417.1598. The single crystals of (2*R*)-**3g** were obtained by slow evaporation from a mixture of acetone and petroleum ether. The supplementary crystallographic data for (2*R*)-**3g** has been deposited in the Cambridge Crystallographic Data Centre (CCDC, http://www.ccdc.cam.ac.uk/) under deposition number 1883470.

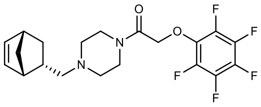

The ^{1}H and ^{13}C NMR spectrum data of (2*S*)-**3g** were identical to **3g**. (2*S*)-**3g** was obtained in a purity of 98.9% (retention time 39.5 min), 97.9% ee. $[\alpha]_D^{20}$: -42.0 (*c* 1.0 in $CHCl_3$). HRMS (ESI): calcd for $C_{20}H_{22}F_5N_2O_2$ $[M+H]^+$: 417.1601, found: 417.1597.

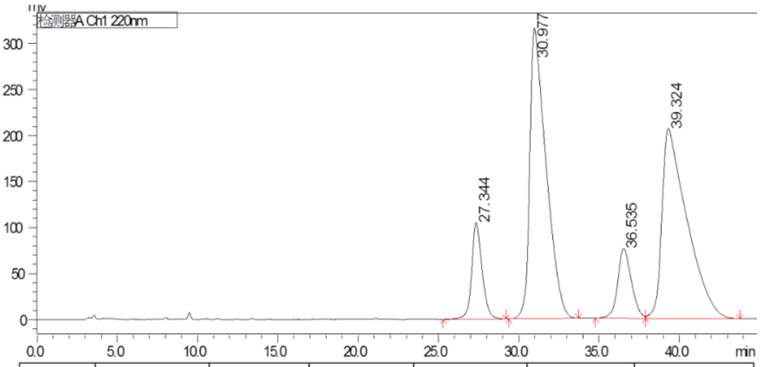

Peak#	Ret. Time	Area	Area%	T.Plate#	Tailing F.	Resolution
1	27.344	4707255	8.733	8867	1.162	--
2	30.977	22269090	41.313	3951	1.778	2.319
3	36.535	4488987	8.328	9096	1.139	3.173
4	39.324	22438196	41.627	2642	2.092	1.215

Analysis of **3g** by analytical HPLC.

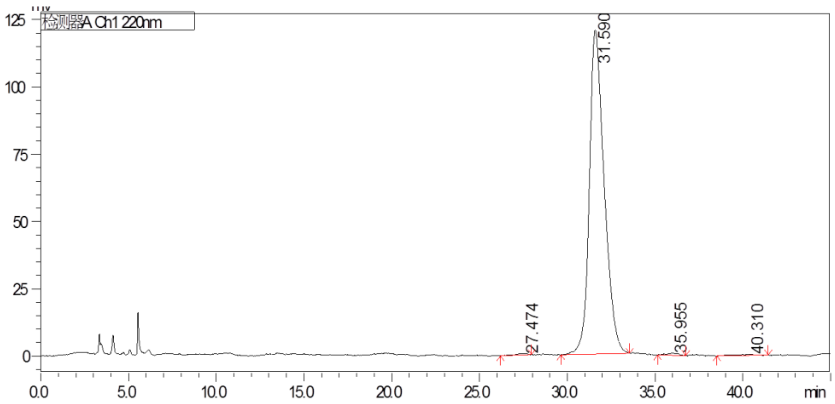

Peak#	Ret. Time	Area	Area%	T.Plate#	Tailing F.	Resolution
1	27.474	20996	0.293	20308	0.691	--
2	31.590	7082145	98.798	6627	1.280	3.543
3	35.955	38299	0.534	29156	0.914	3.646
4	40.310	26852	0.375	51960	0.751	5.620

HPLC analysis of (2*R*)-**3f**.

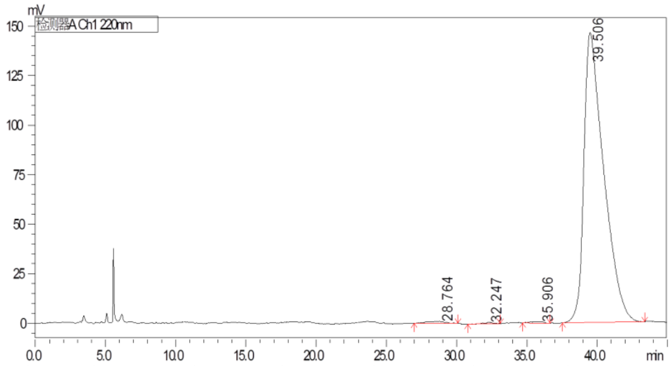

Peak#	Ret. Time	Area	Area%	T.Plate#	Tailing F.	Resolution
1	28.764	75897	0.523	8719	0.768	--
2	32.247	26912	0.185	52207	1.002	3.877
3	35.906	51632	0.356	11516	0.758	3.846
4	39.506	14357154	98.936	3466	1.723	1.790

HPLC analysis of (2*S*)-**3f**.

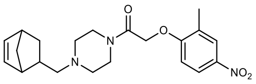

Compound **3h** was synthesized by using the similar methods as **3b**. Yield, 87%. ^{1}H NMR (400 MHz, $CDCl_3$) δ 8.13 – 8.03 (m, 2H), 6.94 – 6.86 (m, 1H), 6.18 – 5.88 (m, 2H), 4.83 (s, 2H), 3.64 (s, 2H), 3.57 (s, 2H), 2.84 (s, 1H), 2.79 (s, 1H), 2.53 – 2.30 (m, 7H), 2.29-2.20 (m, 1H), 2.20 – 2.08 (m, 1H), 2.07-1.95 (m, 1H), 1.85 (t, J = 8.0 Hz, 1H), 1.42 (d, J = 7.6 Hz, 1H), 1.23 (d, J = 8.0 Hz, 1H), 0.56 (d, J = 11.6 Hz, 1H). ^{13}C NMR (101 MHz, $CDCl_3$) δ 164.98, 160.96, 141.51, 137.13, 136.74, 136.44, 132.30, 127.90, 126.16, 123.45, 110.49, 67.55, 64.30, 62.87, 53.54, 52.87, 49.35, 45.19, 45.16, 44.96,

44.88, 42.34, 42.09, 41.75, 38.52, 36.11, 36.03, 31.68, 31.28, 16.33. HRMS (ESI): calcd for $C_{21}H_{28}N_3O_4$ [M+H]$^+$: 386.2080, found: 386.2077.

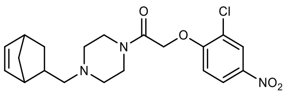

Compound **3i** was synthesized by using the similar methods as **3b**. Yield, 78%. ^{1}H NMR (400 MHz, $CDCl_3$) δ 8.31 (d, J = 2.8 Hz, 1H), 8.14 (dd, J = 8.8, 2.8 Hz, 1H), 7.09 (d, J = 9.2 Hz, 1H), 6.17 – 5.88 (m, 2H), 4.91 (s, 2H), 3.61 (s, 4H), 2.83 (s, 1H), 2.78 (s, 1H), 2.54-2.20 (m, 5H), 2.20 – 2.05 (m, 1H), 2.08 – 1.93 (m, 1H), 1.91 – 1.79 (m, 1H), 1.41 (d, J = 8.4 Hz, 1H), 1.23 (d, J = 8.0 Hz, 1H), 0.60 – 0.50 (m, 1H). ^{13}C NMR (101 MHz, $CDCl_3$) δ 164.28, 158.35, 141.74, 137.15, 136.75, 136.46, 132.31, 126.09, 123.90, 123.35, 112.50, 68.33, 64.29, 62.85, 53.54, 52.88, 49.36, 45.33, 45.17, 44.96, 44.89, 42.36, 42.22, 41.76, 36.11, 36.04, 31.69, 31.28. HRMS (ESI): calcd for $C_{20}H_{25}ClN_3O_4$ [M+H]$^+$: 406.1534, found: 406.1532.

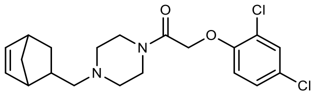

Compound **3j** was synthesized by using the similar methods as **3b**. Yield, 89%. ^{1}H NMR (400 MHz, $CDCl_3$) δ 7.38 (d, J = 2.0 Hz, 1H), 7.18 (dd, J = 8.8, 2.0 Hz, 1H), 6.95 (d, J = 8.8 Hz, 1H), 6.19 – 5.87 (m, 2H), 4.75 (s, 2H), 3.64 (brs, 4H), 2.91 – 2.73 (m, 2H), 2.42 (s, 6H), 1.86 (s, 1H), 1.48 – 1.15 (m, 3H), 0.57 (d, J = 11.6 Hz, 1H). ^{13}C NMR (101 MHz, $CDCl_3$) δ 165.30, 152.13, 137.15, 136.76, 136.52, 132.37, 130.13, 127.73, 126.71, 123.55, 114.38, 68.69, 64.36, 62.92, 53.63, 52.95, 49.40, 45.41, 45.21, 45.04, 44.96, 42.39, 42.17, 41.79, 36.13, 36.05, 31.77, 31.39. HRMS (ESI): calcd for $C_{20}H_{25}Cl_2N_2O_2$ [M+H]$^+$: 395.1293, found: 395.1290.

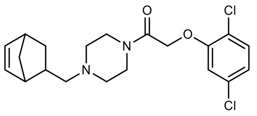

Compound **3k** was synthesized by using the similar methods as **3b**. Yield, 86%. ^{1}H NMR (400 MHz, $CDCl_3$) δ 7.38 (d, J = 2.4 Hz, 1H), 7.18 (dd, J = 8.8, 2.4 Hz, 1H), 6.95 (d, J = 8.8 Hz, 1H), 6.18 –

5.87 (m, 2H), 4.76 (s, 2H), 3.63 (brs, 4H), 2.84 (brs, 1H), 2.79 (brs, 1H), 2.54-2.31 (m, 4H), 2.31-2.21 (m, 1H), 2.20-2.09 (m, 1H), 2.07-1.95 (m, 1H), 1.90 – 1.79 (m, 1H), 1.45 – 1.38 (m, 1H), 1.27 – 1.20 (m, 1H), 0.61 – 0.49 (m, 1H). ^{13}C NMR (101 MHz, $CDCl_3$) δ 165.26, 152.11, 137.11, 136.73, 136.50, 132.36, 130.09, 127.70, 126.66, 123.52, 114.36, 68.64, 64.34, 62.89, 53.60, 52.93, 49.38, 45.39, 45.18, 45.01, 44.93, 42.37, 42.15, 41.78, 36.11, 36.03, 31.74, 31.35. HRMS (ESI): calcd for $C_{20}H_{25}Cl_2N_2O_2$ [M+H]$^+$: 395.1293, found: 395.1290.

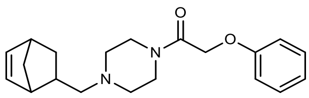

Compound **3l** was synthesized by using the similar methods as **3b**. Yield, 79%. ^{1}H NMR (400 MHz, $CDCl_3$) δ 7.32 – 7.27 (m, 2H), 6.99 (t, J = 7.2 Hz, 1H), 6.97 – 6.92 (m, 2H), 6.17 – 5.88 (m, 2H), 4.68 (s, 2H), 3.63 (s, 2H), 3.59 (s, 2H), 2.90 – 2.76 (m, 2H), 2.53 – 1.79 (m, 8H), 1.41 (dd, J = 8.0, 1.6 Hz, 1H), 1.23 (d, J = 8.0 Hz, 1H), 0.56 (dd, J = 8.4, 3.2 Hz, 1H). ^{13}C NMR (101 MHz, $CDCl_3$) δ 166.25, 157.82, 137.09, 136.72, 136.52, 132.38, 129.53, 121.54, 114.58, 67.65, 64.39, 62.95, 53.60, 52.98, 49.39, 45.33, 45.18, 45.03, 44.96, 42.38, 42.08, 41.78, 36.15, 36.06, 31.76, 31.38. HRMS (ESI): calcd for $C_{20}H_{27}N_2O_2$ [M+H]$^+$: 327.2073, found: 327.2068.

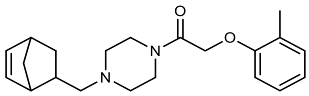

Compound **3m** was synthesized by using the similar methods as **3b**. Yield, 81%. ^{1}H NMR (400 MHz, $CDCl_3$) δ 7.19 – 7.10 (m, 2H), 6.89 (t, J = 7.2 Hz, 1H), 6.87 – 6.82 (m, 1H), 6.19 – 5.87 (m, 2H), 4.69 (s, 2H), 3.62 (s, 4H), 2.90 – 2.73 (m, 2H), 2.43 (d, J = 4.4 Hz, 2H), 2.39 – 2.31 (m, 2H), 2.26 (d, J = 8.6 Hz, 4H), 2.11 (dd, J = 16.0, 8.4 Hz, 1H), 2.05-1.93 (m, 1H), 1.89-1.75 (m, 1H), 1.47 – 1.36 (m, 1H), 1.22 (d, J = 8.0 Hz, 1H), 0.64-0.49 (m, 1H). ^{13}C NMR (101 MHz, $CDCl_3$) δ 166.39, 155.88, 137.07, 136.70, 136.51, 132.38, 130.81, 126.89, 126.54, 121.15, 110.91, 67.83, 64.40, 62.96, 53.68,

52.98, 49.37, 45.38, 45.18, 45.00, 44.94, 42.37, 42.06, 41.77, 36.15, 36.05, 31.74, 31.36, 16.22. HRMS (ESI): calcd for $C_{21}H_{29}N_2O_2$ $[M+H]^+$: 341.2229, found: 341.2226.

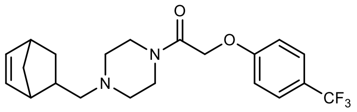

Compound **3n** was synthesized by using the similar methods as **3b**. Yield, 82%, ^{1}H NMR (400 MHz, $CDCl_3$) δ 7.55 (m, 2H), 7.02 (m, 2H), 6.18 – 5.89 (m, 2H), 4.74 (s, 2H), 3.63 (s, 2H), 3.56 (s, 2H), 2.91 – 2.72 (m, 2H), 2.54 – 2.30 (m, 4H), 2.31 – 2.19 (m, 1H), 2.22 – 2.08 (m, 1H), 2.7-1.95 (m, 1H), 1.89 – 1.80 (m, 1H), 1.41 (dd, *J* = 8.0, 1.6 Hz, 1H), 1.23 (d, *J* = 8.4 Hz, 1H), 0.61 – 0.50 (m, 1H). ^{13}C NMR (101 MHz, $CDCl_3$) δ 165.49, 160.28, 137.14, 136.76, 136.50, 132.35, 127.00 (q, *J* = 3.7 Hz). 124.22 (q, *J* = 272.3 Hz), 123.70 (q, *J* = 32.6 Hz), 67.37, 64.36, 62.93, 53.57, 52.93, 49.39, 45.25, 45.19, 45.01, 44.94, 42.38, 42.12, 41.79, 36.15, 36.07, 31.73, 31.34. HRMS (ESI): calcd for $C_{21}H_{26}F_3N_2O_2$ $[M+H]^+$: 395.1946, found: 395.1942.

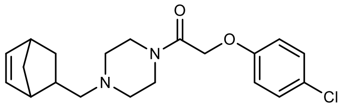

Compound **3o** was synthesized by using the similar methods as **3b**. Yield, 83%. ^{1}H NMR (400 MHz, $CDCl_3$) δ 7.26 – 7.21 (m, 2H), 6.91 – 6.86 (m, 2H), 6.18 – 5.88 (m, 2H), 4.67 (s, 2H), 3.62 (s, 2H), 3.56 (s, 2H), 2.91 – 2.74 (m, 2H), 2.54-2.19 (m, 5H), 2.17-2.06 (m, 1H), 2.05-1.94 (m, 1H), 1.91-1.78 (m, 1H), 1.41 (dd, *J* = 8.0, 1.6 Hz, 1H), 1.23 (d, *J* = 8.4 Hz, 1H), 0.56 (d, *J* = 11.6 Hz, 1H). ^{13}C NMR (101 MHz, $CDCl_3$) δ 165.83, 156.46, 137.10, 136.73, 136.49, 132.36, 129.41, 126.48, 115.92, 67.74, 64.36, 62.92, 53.58, 52.94, 49.37, 45.25, 45.18, 45.00, 44.93, 42.37, 42.07, 41.77, 36.14, 36.05, 31.74, 31.35. HRMS (ESI): calcd for $C_{20}H_{26}ClN_2O_2$ $[M+H]^+$: 361.1683, found: 361.1680.

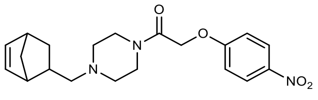

Compound **3p** was synthesized by using the similar methods as **3b**. Yield, 82%. ^{1}H NMR (400 MHz,

CDCl3) δ 8.27 – 8.15 (m, 2H), 7.06 – 7.00 (m, 2H), 6.18 – 5.88 (m, 2H), 4.81 (s, 2H), 3.64 (s, 2H), 3.55 (s, 2H), 2.92 – 2.76 (m, 2H), 2.456-2.31 (m, 4H), 2.31 – 2.21 (m, 1H), 2.20 – 2.09 (m, 1H), 2.08 – 1.94 (m, 1H), 1.92-1.78 (m, 1H), 1.42 (d, J = 7.8 Hz, 1H), 1.23 (d, J = 8.0 Hz, 1H), 0.57 (d, J = 11.6 Hz, 1H). ^{13}C NMR (101 MHz, CDCl3) δ 164.81, 162.82, 142.03, 137.10, 136.73, 136.44, 132.29, 125.84, 114.80, 114.78, 114.72, 114.68, 114.66, 67.35, 64.29, 62.86, 53.50, 52.87, 49.35, 45.16, 44.95, 44.87, 42.34, 42.12, 41.74, 36.12, 36.05, 31.68, 31.27. HRMS (ESI): calcd for $C_{20}H_{26}N_3O_4$ $[M+H]^+$: 372.1923, found: 372.1925.

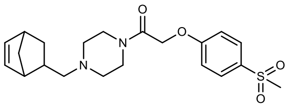

Compound **3q** was synthesized by using the similar methods as **3b**. Yield, 79%. ^{1}H NMR (400 MHz, CDCl3) δ 7.90 – 7.85 (m, 2H), 7.12 – 7.05 (m, 2H), 6.20 – 5.88 (m, 2H), 4.79 (s, 2H), 3.64 (s, 2H), 3.55 (s, 2H), 3.03 (s, 3H), 2.84 (s, 1H), 2.79 (s, 1H), 2.58-2.32 (m, 4H), 2.31-2.21 (m, 1H), 2.18-2.08 (s, 1H), 2.06-1.96 (s, 1H), 1.90 – 1.80 (m, 1H), 1.41 (dd, J = 8.0, 2.0 Hz, 1H), 1.24 (d, J = 6.4 Hz, 1H), 0.57 (dd, J = 8.4, 2.8 Hz, 1H). ^{13}C NMR (101 MHz, CDCl3) δ 165.00, 161.93, 137.08, 136.70, 136.42, 133.05, 132.28, 129.50, 115.11, 67.01, 64.26, 62.84, 53.46, 52.84, 49.31, 45.12, 45.07, 44.92, 44.84, 44.69, 42.30, 42.03, 41.71, 36.07, 35.99, 31.64, 31.25. HRMS (ESI): calcd for $C_{21}H_{29}N_2O_4S$ $[M+H]^+$: 405.1848, found: 405.1845.

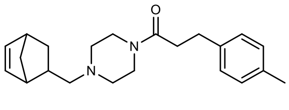

Compound **3r** was synthesized by using the similar methods as **3b**. Yield, 86%. ^{1}H NMR (400 MHz, CDCl3) δ 7.14 – 7.05 (m, 4H), 6.16 – 5.89 (m, 2H), 3.62 (s, 2H), 3.38 (s, 2H), 2.98 – 2.88 (m, 2H), 2.87 – 2.72 (m, 2H), 2.63 – 2.52 (m, 2H), 2.43 – 2.21 (m, 7H), 2.17-2.07 (m, 1H), 1.97 (dd, J = 12.4, 7.2 Hz, 1H), 1.89 – 1.78 (m, 1H), 1.45 – 1.37 (m, 1H), 1.35 – 1.15 (m, 2H), 0.62 – 0.48 (m, 1H). ^{13}C NMR (101 MHz, CDCl3) δ 170.50, 138.10, 137.03, 136.67, 136.50, 135.50, 132.36, 129.08, 128.25,

128.22, 128.20, 64.45, 63.01, 53.46, 53.03, 49.35, 45.47, 45.17, 45.04, 44.97, 42.35, 41.74, 41.52, 36.13, 36.01, 35.08, 31.80, 31.44, 31.01, 20.92. HRMS (ESI): calcd for $C_{22}H_{31}N_2O$ $[M+H]^+$: 339.2436, found: 339.2435.

Supplementary Note. Synthesis of compound **4a**.

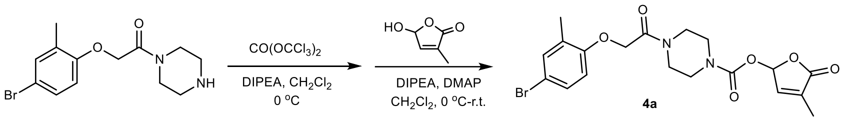

To a solution of 2-(4-bromo-2-methylphenoxy)-1-(piperazin-1-yl)ethan-1-one (0.66 g, 2.1 mmol) and DIPEA (0.54 g, 4.2 mmol) in CH_2Cl_2 (20 mL) was added to a solution of triphosgene (0.78 g, 2.64 mmol) in CH_2Cl_2 (25 mL) at 0 °C for 15 min. After stirring at this temperature for 2 h, the solvent was removed under reduced pressure, the residues was again dissolved in CH_2Cl_2 (50 mL). 5-hydroxy-3-methylfuran-2(5*H*)-one (0.36 g, 3.16 mmol), DIPEA (0.82 g, 6.3 mmol), and DMAP (26 mg, 0.21 mmol) were added to the solution at 0 °C. After stirring at 0 °C for another 2 h, the reaction solution was slowly warmed to room temperature and reacted for another 10 h. After completing the reaction check by TLC ($V_{\text{petroleum ether}}$: V_{acetone} = 20 : 7) detection, water (30 mL) was added to the reaction solution and stirred vigorously for 15 min. The organic layer was separated and washed with saturated NaCl solution (100 mL) and concentrated to dryness. The cure product was first purified by flash chromatography on silica gel and then recrystallized from diethyl ether to afford pure compound **4a** (0.66 g, yield 69%). ^{1}H NMR (400 MHz, CD_2Cl_2) δ 7.28 (s, 1H), 7.25 (dd, *J* = 8.8, 2.4 Hz, 1H), 6.93 – 6.88 (m, 1H), 6.84 (s, 1H), 6.72 (d, *J* = 8.8 Hz, 1H), 4.70 (s, 2H), 3.71 – 3.37 (m, 8H), 2.22 (s, 3H), 1.95 (t, *J* = 1.2 Hz, 3H). ^{13}C NMR (101 MHz, $CDCl_3$) δ 170.88, 166.25, 154.67, 152.38, 152.23, 141.90, 134.21, 133.36, 129.35, 128.69, 113.35, 112.40, 93.51, 67.73, 67.59, 44.75, 44.04, 43.81,

43.60, 43.36, 41.40, 15.93, 10.43. HRMS (ESI) Calcd for $C_{19}H_{21}BrN_2NaO_6$ $[M + Na]^+$: 475.0481; found: 475.0478.

Supplementary Note. Separation of the enantiomers of **4a**.

The chiral isomers of **4a** were analyzed by the Shimadzu LC-20AD CP-HPLC-08 instrument with a CHIRALPAK IH (0.46 cm I.D. × 25 cm L) chiral column. Parameters set for chiral HPLC used for analysis the isomers were as follows: temperature: 25 °C; wavelength: UV 210 nm; mobile phase: $V_{CH2Cl2}/V_{ethyl\ acetate}$ = 85/5; injection volume: 5.0 μL; flow rate: 1.0 mL/min. Two isomers were found in **4a**, the retention times for isomer a ((*S*)-**4a**) was 6.4 min, and for isomer b ((***R***)-**4a**) was 7.6 min. The separation of two isomers was performed on the YMC-1 (K-PREP-K-100) preparative HPLC instrument with CHIRALPAK IF 50 ×250 mm, 5 μm column. The parameters set for preparative HPLC were as follows: temperature: 25 °C; detection wavelength: UV 214 nm; mobile phase: $V_{CH2Cl2}/V_{ethyl\ acetate}$ = 85/5; flow rate: 60 mL/min; injection volume: 3 mL. In the separation process, each peak was collected independently, and the combined solvent of each peak was removed by rotary evaporation at 35 °C. The final purity of (*S*)-**4a** and (***R***)-**4a** was checked by again analysis HPLC.

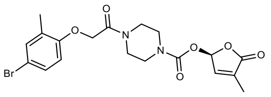

The ^1H and ^{13}C NMR spectrum data of (*R*)-**4a** were identical to **4a**. (*R*)-**4a** was obtained in a purity of above 99% (retention time 7.48 min), >99 % ee. $[\alpha]_D^{20}$: 48.8 (*c* 0.5 in CH_3Cl). HRMS (ESI) Calcd for $C_{19}H_{21}BrN_2NaO_6$ $[M + Na]^+$: 475.0481; found: 475.0479. The absolute structure of (*R*)-**4a** was confirmed by comparing the experimental ECD spectrum with the calculated spectrum (results shown below).

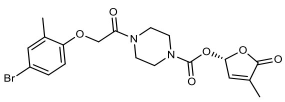

The ^{1}H and ^{13}C NMR spectrum data of (*S*)-**4a** were identical to **4a**. (*S*)-**4b** was obtained in a purity of above 99 % (retention time 6.36 min), >99% ee. $[\alpha]_D^{20}$: 48.0 (*c* 0.8 in CH_3Cl). HRMS (ESI) Calcd for $C_{19}H_{21}BrN_2NaO_6$ [M + Na]$^+$: 475.0481; found: 475.0478. The absolute structure of (*R*)-**4a** was confirmed by comparing the experimental ECD spectrum with the calculated spectrum (results shown below).

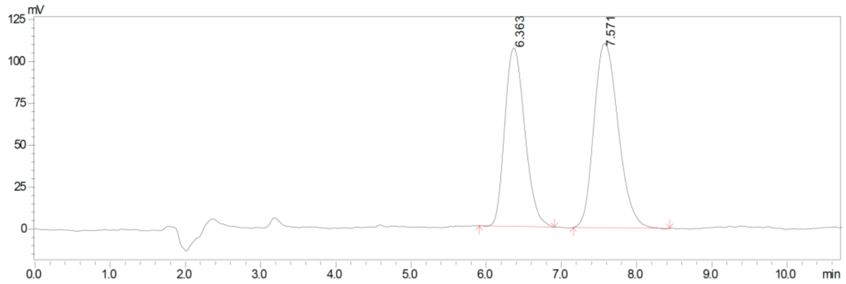

Peak#	Ret. Time	Area	T.Plate#	Tailing F.	Area%
1	6.363	1974054	2736.803	1.251	44.3763
2	7.571	2474388	2649.665	1.254	55.6237

Analysis of **4a** by analytical HPLC.

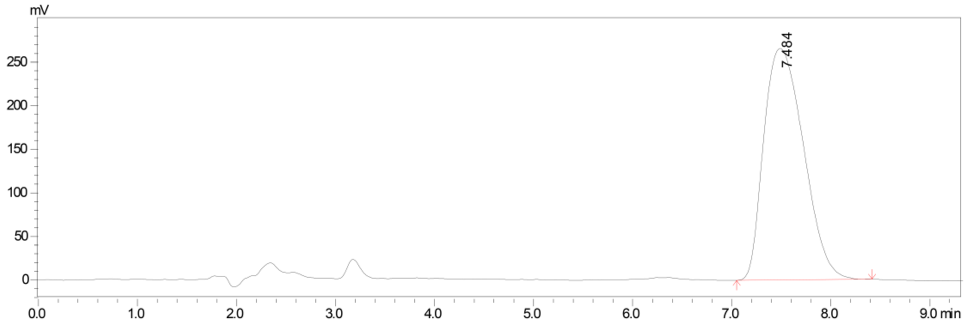

Peak#	Ret. Time	Area	T.Plate#	Tailing F.	Area%
1	7.484	7433623	1605.050	1.371	100.00

HPLC analysis of (*R*)-**4a**.

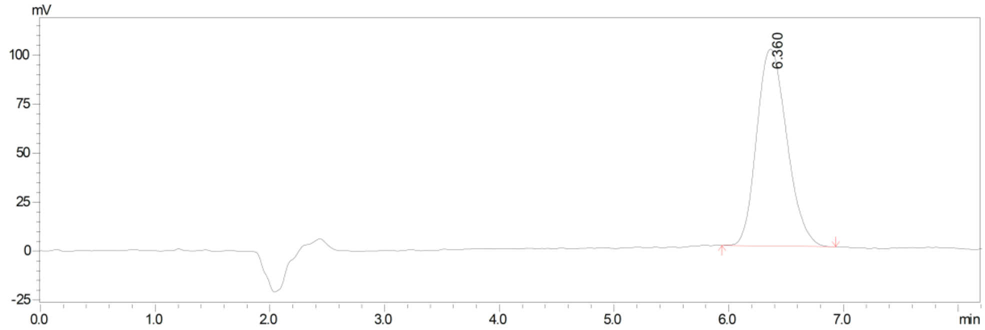

Peak#	Ret. Time	Area	T.Plate#	Tailing F.	Area%
1	6.360	1841045	2743.744	1.208	100.00

HPLC analysis of (*S*)-**4a**.

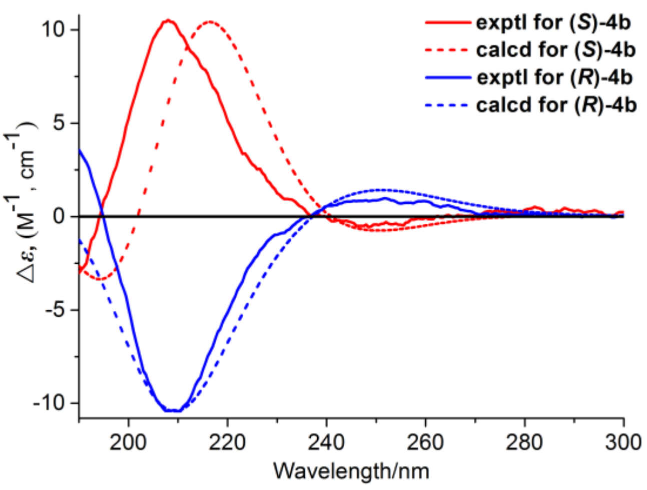

Comparison of the experimental ECD spectrum of (*R*)-**4b** and (*S*)-**4b** with their corresponding calculated spectrum. ECD values were calculated by Gaussian 09 at apfd/6-311+g(2d,p) level[34].

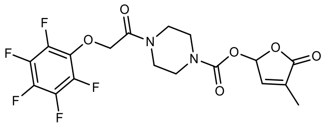

Compound **4b** was synthesized by using the similar methods as **4a**. Yield, 81%. ^{1}H NMR (400 MHz, $CDCl_3$) δ 6.92 (s, 2H), 4.95 – 4.78 (m, 2H), 3.83 – 3.39 (m, 8H), 2.00 (s, 3H). ^{13}C NMR (101 MHz, $CDCl_3$) δ 170.94, 165.10, 165.01, 152.43, 152.34, 142.41 (m, 1C), 141.95, 139.94 (m, 1C), 138.83 (m, 1.5 C), 136.40 (m, 1.5 C), 132.4 6 (m, 1 C), 71.36, 71.18, 44.33, 43.74, 43.48, 43.22, 41.19, 10.36. HRMS (ESI) Calcd for $C_{18}H_{15}F_5N_2NaO_6$ $[M + Na]^+$: 473.0748; found: 473.0745.

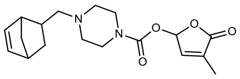

Compound **4c** was synthesized by using the similar methods as **4a**. Yield 58%. ^{1}H NMR (400 MHz, $CDCl_3$) δ 6.91 (s, 2H), 6.17-5.87 (m, 2H), 3.65-3.34 (m, 4H), 2.81 (d, J = 24.4 Hz, 2H), 2.54 – 2.18 (m, 5H), 2.20 – 2.07 (m, 1H), 2.06 – 1.93 (m, 4H), 1.91 – 1.76 (m, 1H), 1.41 (d, J = 7.6 Hz, 1H), 1.23 (d, J = 8.0 Hz, 1H), 0.56 (d, J = 11.6 Hz, 1H). ^{13}C NMR (101 MHz, $CDCl_3$) δ 171.17, 152.44, 142.33, 136.97, 136.61, 136.43, 134.02, 132.31, 93.58, 62.92, 52.91, 52.87, 52.73, 52.69, 49.28, 44.91, 43.95, 43.86, 42.27, 36.06, 31.28, 10.48. HRMS (ESI) Calcd for $C_{18}H_{25}N_2O_4$ $[M + H]^+$: 333.1814; found: 333.1812.

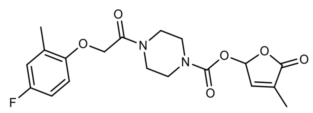

Compound **4d** was synthesized by using the similar methods as **4a**. Yield, 90%. ^{1}H NMR (400 MHz, $CDCl_3$) δ 6.94 – 6.74 (m, 5H), 4.70 (s, 2H), 3.85 – 3.32 (m, 8H), 2.23 (s, 3H), 1.99 (s, 3H). ^{13}C NMR (101 MHz, $CDCl_3$) δ 170.90, 166.55, 157.10 (d, J = 239.3 Hz), 152.43, 152.30, 151.69, 141.92, 134.23, 128.29, 117.53 (d, J = 22.7 Hz), 112.44 (d, J = 23.2 Hz), 111.76 (d, J = 8.6 Hz), 93.56, 68.11, 44.76, 44.06, 43.85, 43.63, 43.41, 41.41, 16.17, s10.40. HRMS (ESI) Calcd for $C_{19}H_{21}FN_2NaO_6$ $[M + Na]^+$: 415.1281; found: 415.1280.

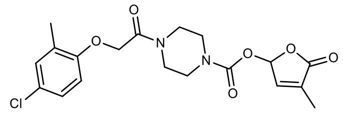

Compound **4e** was synthesized by using the similar methods as **4a**. Yield, 80%. ^{1}H NMR (400 MHz, $CDCl_3$) δ 7.13 (s, 1H), 7.10 (d, J = 8.8 Hz, 1H), 6.90 (s, 2H), 6.78 (d, J = 8.4 Hz, 1H), 4.71 (s, 2H), 3.81 – 3.32 (m, 8H), 2.21 (s, 3H), 1.99 (s, 3H). ^{13}C NMR (101 MHz, $CDCl_3$) δ 170.93, 166.42, 154.23, 152.49, 152.35, 141.91, 134.37, 130.68, 128.31, 126.48, 126.07, 111.98, 93.63, 67.85, 44.86, 44.13, 43.93, 43.71, 43.49, 41.53, 16.06, 10.50. HRMS (ESI) Calcd for $C_{19}H_{21}ClN_2NaO_6$ $[M + Na]^+$: 431.0986; found: 431.0985.

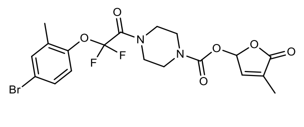

Compound **4f** was synthesized by using the similar methods as **4a**. Yield, 59%. ^{1}H NMR (400 MHz, $CDCl_3$) δ 7.39 (d, J = 1.6 Hz, 1H), 7.32 (d, J = 8.4 Hz, 1H), 7.09 (t, J = 8.8 Hz, 1H), 6.91 (s, 2H), 3.92 – 3.43 (m, 8H), 2.26 (s, 3H), 2.00 (s, 3H). ^{13}C NMR (101 MHz, $CDCl_3$) δ 170.91, 157.85 (t, J = 33.6 Hz), 157.48, 152.48, 146.94, 141.88, 134.51, 134.24, 132.98, 129.83, 122.59, 119.21, 115.20 (t,

J = 276.0 Hz), 93.72, 45.65, 44.12, 43.87, 43.57, 43.33, 42.78, 16.33, 10.55. HRMS (ESI) Calcd for $C_{19}H_{19}BrF_2N_2NaO_6$ [M + Na]$^+$: 511.0292; found: 511.0290.

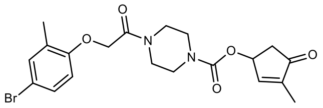

Compound **4g** was synthesized by using the similar methods as **4a**. Yield, 38%. ^{1}H NMR (400 MHz, CD_2Cl_2) δ 7.28 (s, 1H), 7.26 – 7.19 (m, 2H), 6.72 (d, J = 8.8 Hz, 1H), 5.73 – 5.63 (m, 1H), 4.70 (s, 2H), 3.56 (s, 4H), 3.44 (s, 4H), 2.81 (dd, J = 18.8, 6.4 Hz, 1H), 2.33 (dd, J = 18.8, 2.0 Hz, 1H), 2.21 (s, 3H), 1.87 – 1.76 (m, 3H). ^{13}C NMR (101 MHz, CD_2Cl_2) δ 205.25, 166.53, 155.57, 153.24, 145.62, 133.80, 129.78, 129.54, 113.59, 113.09, 71.92, 68.12, 45.33, 44.22, 44.14, 44.01, 42.00, 41.81, 16.24, 10.12. HRMS (ESI) Calcd for $C_{20}H_{23}BrN_2NaO_5$ [M + Na]$^+$: 473.0688; found: 473.0685.

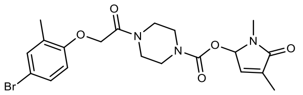

Compound **4h** was synthesized by using the similar methods as **4a**. Yield, 50%. ^{1}H NMR (400 MHz, $CDCl_3$) δ 7.28 – 7.16 (m, 2H), 6.73 (d, J = 8.4 Hz, 1H), 6.56 (s, 1H), 6.28 (s, 1H), 4.71 (s, 2H), 3.64 (s, 2H), 3.61 (s, 2H), 3.51 (s, 2H), 3.44 (s, 2H), 2.94 (s, 3H), 2.21 (s, 3H), 1.92 (s, 3H). ^{13}C NMR (101 MHz, $CDCl_3$) δ 170.30, 166.11, 154.56, 137.96, 134.67, 133.21, 129.20, 128.53, 113.20, 112.28, 83.65, 67.49, 44.66, 43.80, 43.55, 43.37, 43.06, 41.36, 26.72, 15.77, 10.63. HRMS (ESI) Calcd for $C_{20}H_{24}BrN_3NaO_5$ [M + Na]$^+$: 488.0797; found: 488.0795.

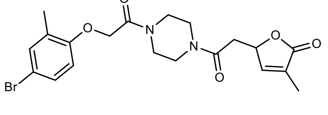

Compound **4i** was synthesized by using the similar methods as **3a**. Yield, 83%. ^{1}H NMR (400 MHz, CD_2Cl_2) δ 7.36-7.18 (m, 3H), 6.72 (d, J = 8.4 Hz, 1H), 5.32 (s, 1H), 4.71 (s, 2H), 3.76-3.52 (m, 6H), 3.52-3.33 (m, 2H), 2.84 (dd, J = 16.0, 6.8 Hz, 1H), 2.57 (dd, J = 16.0, 6.8 Hz, 1H), 2.21 (s, 3H), 1.87 (s, 3H). ^{13}C NMR (101 MHz, CD_2Cl_2) δ 174.03, 167.69, 166.76, 155.55, 149.52, 149.43, 133.82,

130.29, 129.81, 129.62, 113.68, 113.25, 78.11, 78.06, 68.11, 68.00, 45.91, 45.48, 45.30, 45.18, 42.09, 41.93, 41.51, 37.28, 37.20, 16.23, 10.68. HRMS (ESI) Calcd for $C_{20}H_{23}BrN_2NaO_5$ $[M + Na]^+$: 473.0688; found: 473.0684.

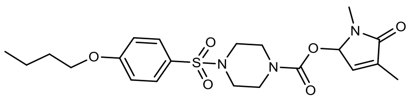

Compound **4j** was synthesized by using the similar methods as **4a**. Yield, 36%. ^{1}H NMR (400 MHz, $CDCl_3$) δ 7.67 (m, 2H), 7.00 (m, 2H), 6.51 (t, J = 2.0 Hz, 1H), 6.21 (s, 1H), 4.02 (t, J = 6.4 Hz, 2H), 3.60 (s, 2H), 3.54 (s, 2H), 3.01 (s, 2H), 2.96 (s, 2H), 2.88 (s, 3H), 1.90 (s, 3H), 1.80 (dt, J = 14.4, 6.4 Hz, 2H), 1.58 – 1.44 (m, 2H), 0.99 (t, J = 7.2 Hz, 3H). ^{13}C NMR (101 MHz, $CDCl_3$) δ 170.47, 162.76, 153.60, 138.13, 134.75, 129.65, 126.17, 114.62, 68.02, 45.82, 45.52, 43.19, 42.91, 30.81, 26.86, 18.94, 13.59, 10.77. HRMS (ESI) Calcd for $C_{21}H_{29}N_3NaO_6S$ $[M + Na]^+$: 474.1675; found: 474.1673.

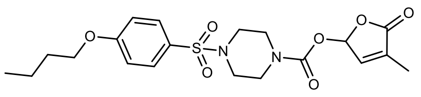

Compound **SPL7** was synthesized by using the similar methods as **4a**. Yield 86%. ^{1}H NMR (400 MHz, $CDCl_3$) δ 7.65 (m, 2H), 6.99 (m, 2H), 6.85 (s, 1 Hz, 1H), 6.83 (s, 1H), 4.03 (t, J = 6.0 Hz, 2H), 3.76 (d, J = 12.8 Hz, 1H), 3.66 (d, J = 12.8Hz, 1H), 3.56 – 3.40 (m, 2H), 3.28 – 3.08 (m, 2H), 2.89 – 2.73 (m, 2H), 1.97 (s, 3H), 1.85 – 1.74 (m, 2H), 1.60 – 1.44 (m, 2H), 1.04-0.94 (m, 3H). ^{13}C NMR (101 MHz, $CDCl_3$) δ 170.97, 162.93, 152.15, 141.96, 134.32, 129.69, 126.05, 114.76, 93.60, 68.12, 45.56, 43.38, 43.14, 30.90, 19.03, 13.67, 10.49. HRMS (ESI) Calcd for $C_{20}H_{26}N_2NaO_7S$ $[M + Na]^+$: 461.1358; found: 461.1355.

Supplementary Note. Synthesis of compound Ac-his-ome-D.

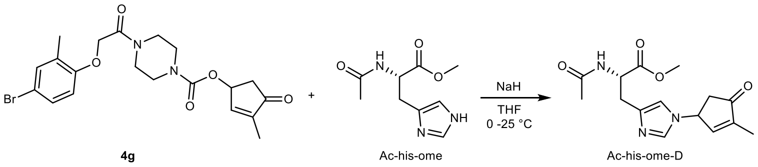

NaH (60%, 1.6 mg, 0.04 mmol) was added to a solution of Ac-his-ome (7 mg, 0.033 mmol) in tetrahydrofuran (THF, 10 mL) at 0 °C under N_2 with stirring. After 10 min, **4g** (15 mg, 0.033 mmol) was added to the mixture. Next, the reaction was moved to room temperature and stirred for six hours. 0.2 mL of the reaction mixture was taken out by a syringe and diluted with 0.8 mL of acetonitrile, and the solution was filtrated and used for the UPLC-HRMS analysis. A new compound with a mass of 306.1435 was detected, which corresponded well with the mass of Ac-his-ome-D (HRMS (ESI): calcd for $C_{15}H_{20}N_3O_4$ [M+H]$^+$: 306.1454).

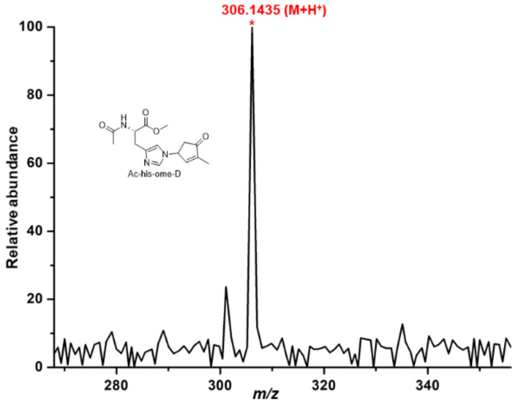

A new compound (m/z = 306.1435) corresponding to Ac-his-ome-D in the reaction solution was detected by HPLC-HRMS analysis.

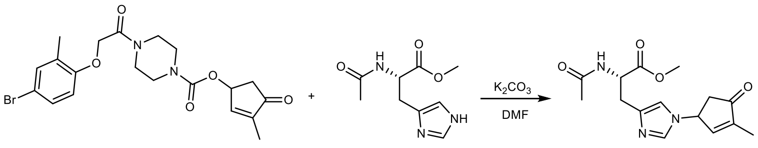

4g Ac-his-ome Ac-his-ome-D

K_2CO_3 (5.7 mg, 0.042 mmol) was added to a solution of Ac-his-ome (7 mg, 0.033 mmol) in *N*,*N*-dimethylformamide (DMF, 10 mL) at 25 °C with stirring. After 10 min, **4g** (15 mg, 0.033 mmol) was added to the mixture. Next, the reaction was stirred for six hours. 0.2 mL of the reaction mixture was taken out by a syringe and diluted with 0.8 mL of acetonitrile, and the solution was filtrated and used for the UPLC-HRMS analysis. A new compound with a mass of 306.1430 was

detected, which corresponded well with the mass of Ac-his-ome-D (HRMS (ESI): calcd for $C_{15}H_{20}N_3O_4$ [M+H]$^+$: 306.1454).

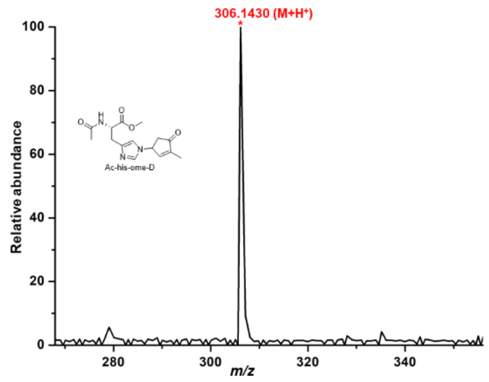

A new compound (*m/z* = 306.1430) corresponding to Ac-his-ome-D in the reaction solution was detected by HPLC-HRMS analysis.

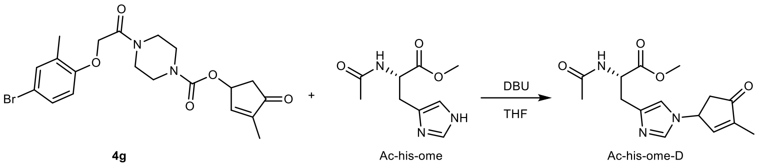

1,8-diazabicyclo[5.4.0]undec-7-ene (DBU, 7.6 mg, 0.05 mmol) was added to a solution of Ac-his-ome (7 mg, 0.033 mmol) in tetrahydrofuran (THF, 10 mL) at 25 °C under. After 10 min, **4g** (15 mg, 0.033 mmol) was added to the mixture. Next, the reaction was moved to room temperature and stirred for six hours. 0.2 mL of the reaction mixture was taken out by a syringe and diluted with 0.8 mL of acetonitrile, and the solution was filtrated and used for the UPLC-HRMS analysis. A new compound with a mass of 306.1436 was detected, which corresponded well with the mass of Ac-his-ome-D (HRMS (ESI): calcd for $C_{15}H_{20}N_3O_4$ [M+H]$^+$: 306.1454).

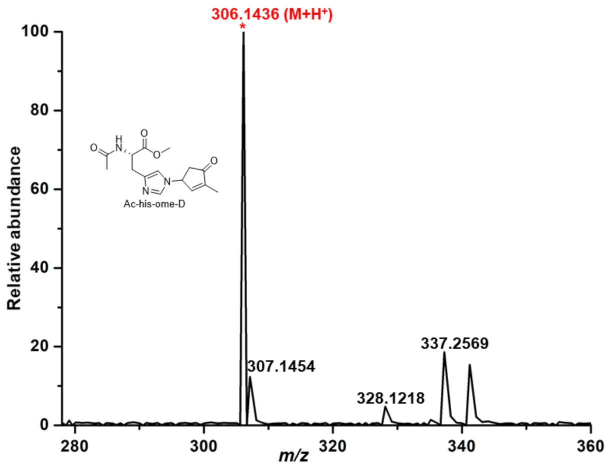

A new compound (*m*/*z* = 306.1436) corresponding to Ac-his-ome-D in the reaction solution was detected by HPLC-HRMS analysis.

References

1. Otwinowski, Z., Minor W. [20]Processing of X-ray diffraction data collected in oscillation mode. In: *Methods in Enzymology*). Academic Press (1997).

2. Emsley, P., Lohkamp, B., Scott, W. G, Cowtan, K. Features and development of Coot. *Acta Crystallogr. D Biol. Crystallogr.* **66**, 486–501 (2010).

3. Morris, G. M. et al. AutoDock4 and AutoDockTools4: Automated docking with selective receptor flexibility. *J. Comput. Chem.* **30**, 2785–2791 (2009).

4. Zhao, L. H. et al. Destabilization of strigolactone receptor DWARF14 by binding of ligand and E3-ligase signaling effector DWARF3. *Cell Res.* **25**, 1219–1236 (2015).

5. Case, D. A. et al. AMBER 14, University of California, San Francisco., (2014).

6. Yang, J. F., Yin, C. Y., Wang, D., Jia, C. Y., Hao, G. F., Yang, G. F. Molecular determinants elucidate the selectivity in abscisic acid receptor and HAB1 protein interactions. *Front Chem.* **8**, 425 (2020).

7. Wang, B. F. et al. Quantitative structural insight into human variegate porphyria disease. *J. Biol. Chem.* **288**, 11731−11740 (2013).

8. DeLano, W. The PyMOL Molecular Graphics System. San Carlos, CA: Delano Scientific., (2002).

9. Frisch, M. J. et al. Gaussian 09, Revision B.01, Gaussian, Inc., Wallingford CT. (2010).

10. Hao, G. F. et al. Computational discovery of picomolar Q_o site inhibitors of cytochrome *bc1* complex. *J. Am. Chem. Soc.* **134**, 11168−11176 (2012).

11. Yao, R. et al. ShHTL7 is a non-canonical receptor for strigolactones in root parasitic weeds. *Cell Res.* **27**, 838–841 (2017).

12. Uraguchi, D. et al. A femtomolar-range suicide germination stimulant for the parasitic plant *Striga hermonthica*. *Science* **362**, 1301−1305 (2018).

13. Casino, P., Miguel-Romero, L., Marina, A. Visualizing autophosphorylation in histidine kinases. *Nat. Commun.* **5**, 3258 (2014).

14. Nie, L. et al. The structural basis of fatty acid elongation by the ELOVL elongases. *Nat. Struct. Mol. Biol.* **28**, 512–520 (2021).

15. Burke, A. J. et al. Design and evolution of an enzyme with a non-canonical organocatalytic mechanism. *Nature* **570**, 219–223 (2019).

16. Morgen, M. et al. Spiroepoxytriazoles are fumagillin-like irreversible inhibitors of MetAP2 with potent cellular activity. *ACS Chem. Biol.* **11**, 1001−1011 (2016).

17. Selvy, P. E., Lavieri, R. R., Lindsley, C. W., Brown, H. A. Phospholipase D: enzymology, functionality, and chemical modulation. *Chem. Rev.* **111**, 6064−6119 (2011).

18. Gehringer, M., Laufer, S. A. Emerging and re-emerging warheads for targeted covalent inhibitors: applications in medicinal chemistry and chemical biology. *J. Med. Chem.* **62**, 5673−5724 (2019).

19. Pluta, R. et al. Structural basis of a histidine-DNA nicking/joining mechanism for gene transfer

and promiscuous spread of antibiotic resistance. *Proc. Natl. Acad. Sci.* **114**, E6526–E6535 (2017).

20. Kuroki, R., Weaver Larry, H., Matthews Brian, W. Structural basis of the conversion of T4 lysozyme into a transglycosidase by reengineering the active site. *Proc. Natl. Acad. Sci.* **96**, 8949–8954 (1999).

21. Shah, R., Maize, K. M., Zhou, X., Finzel, B. C., Wagner, C. R. Caught before released: structural mapping of the reaction trajectory for the sofosbuvir activating enzyme, human histidine triad nucleotide binding protein 1 (hHint1). *Biochemistry* **56**, 3559–3570 (2017).

22. Rothlisberger, D. et al. Kemp elimination catalysts by computational enzyme design. *Nature* **453**, 190–195 (2008).

23. Zhao, L. H. et al. Crystal structures of two phytohormone signal-transducing alpha/beta hydrolases: karrikin-signaling KAI2 and strigolactone-signaling DWARF14. *Cell Res.* **23**, 436–439 (2013).

24. Kagiyama, M. et al. Structures of D14 and D14L in the strigolactone and karrikin signaling pathways. *Genes Cells* **18**, 147–160 (2013).

25. Guo, Y., Zheng, Z., La Clair, J. J., Chory, J., Noel, J. P. Smoke-derived karrikin perception by the α/β-hydrolase KAI2 from *Arabidopsis*. *Proc. Natl. Acad. Sci.* **110**, 8284–8289 (2013).

26. Xu, Y. et al. Structural analysis of HTL and D14 proteins reveals the basis for ligand selectivity in *Striga*. *Nat. Commun.* **9**, 3947 (2018).

27. Toh, S. et al. Structure-function analysis identifies highly sensitive strigolactone receptors in *Striga*. *Science* **350**, 203–207.

28. Shahul Hameed, U. et al. Structural basis for specific inhibition of the highly sensitive ShHTL7 receptor. *EMBO Rep.* **19**, e45619 (2018).

29. Zhang, Y., Wang, D., Shen, Y., Xi, Z. Crystal structure and biochemical characterization of *Striga hermonthica* HYPO-SENSITIVE TO LIGHT 8 (ShHTL8) in strigolactone signaling pathway. *Biochem. Biophys. Res. Commun.* **523**, 1040–1045 (2020).

30. Xu, Y. et al. Structural basis of unique ligand specificity of KAI2-like protein from parasitic weed Striga hermonthica. *Sci. Rep.* **6**, 31386 (2016).

31. Lee, I. et al. A missense allele of KARRIKIN-INSENSITIVE2 impairs ligand-binding and downstream signaling in *Arabidopsis thaliana*. *J. Exp. Bot.* **69**, 3609–3623 (2018).

32. Bythell-Douglas, R., Waters, M. T., Scaffidi, A., Flematti, G. R., Smith, S. M., Bond, C. S. The structure of the karrikin-insensitive protein (KAI2) in *Arabidopsis thaliana*. *PLoS ONE* **8**, e54758 (2013).

33. Burger, M. et al. Structural basis of barrikin and non-natural strigolactone perception in *Physcomitrella patens*. *Cell Rep.* **26**, 855–865 (2019).

34. Jiao, W. H. et al. Dysideanones A–C, unusual sesquiterpene quinones from the south China sea sponge *Dysidea avara*. *J. Nat. Prod.* **77**, 346–350 (2014).
